# Determining the optimal frequency of SARS-CoV-2 regular asymptomatic testing: A randomized feasibility trial in a home care setting

**DOI:** 10.1371/journal.pone.0303344

**Published:** 2024-07-03

**Authors:** Jana Butzmann, Annett Hellriegel-Nehrkorn, Milica Dilas, Robert Pohl, Martin Hellmich, Christian J. Apfelbacher, Achim J. Kaasch

**Affiliations:** 1 Institute of Medical Microbiology and Hospital Hygiene, Medical Faculty, Otto von Guericke University, Magdeburg, Germany; 2 Institute for Social Medicine and Health Systems Research, Medical Faculty, Otto von Guericke University, Magdeburg, Germany; 3 Institute of Medical Statistics and Computational Biology, Faculty of Medicine and University Hospital Cologne, University of Cologne, Cologne, Germany; United States Environmental Protection Agency, UNITED STATES

## Abstract

**Background:**

The SARS-CoV-2 pandemic presented a challenge for caregiving relatives in the home care setting. Caregivers can transmit SARS-CoV-2 to their relatives who are often at high risk for a severe course of COVID-19. Regular testing of asymptomatic caregivers for SARS-CoV-2 may reduce the risk of transmission. The optimal method and frequency of regular asymptomatic testing is unknown. We conducted a prospective, randomised trial to assess the feasibility, recruitment and acceptance of different testing frequencies. This serves to inform a future definitive randomised controlled trial.

**Methods:**

We carried out a parallel three-armed feasibility trial, enrolling adult participants who provided home-based care for a relative at least twice a week. Participants were randomly assigned using sealed envelopes to either conduct saliva-based antigen self-testing at a frequency of once a week (group I), twice a week (group II), or every two days (group III). The participants completed questionnaires on a weekly basis. Main outcome measures were feasibility of recruitment, adherence to self-tests and distress caused by self-testing. We further collected data on the use of mouth-nose mask.

**Results:**

From 25 March to 7 May 2021 we assessed 27 participants and randomised 26 in the study: 8 participants in group I, 8 in group II and 10 in group III. All participants completed the study. In group I 48/48 (100.0%; 95% CI 92.6% to 100.0%), in group II 93/96 (96.9%; 95% CI 91.2% to 98.9%) and in group III 209/210 (99.5%; 95% CI 97.4% to 99.9%) self-tests were carried out at home. Participants did not perceive regular self-testing as burdensome in any of the study arms. We did not observe any infection with SARS-CoV-2. During the study, mask adherence decreased from 35% to 19% in all groups.

**Conclusion:**

Conducting such a study was feasible. The participants tolerated regular self-testing well, which was reflected in a high level of test adherence. However, regular self-testing may have led to decreased protective behaviour. To demonstrate that regular asymptomatic testing reduces infection transmission, a future definitive trial should be performed at a time of a high prevalence of SARS-CoV-2 and be implemented as a multicentre study.

**Trial registration:**

The trial is registered with the German Clinical Trials Register, DRKS00026234.

## Introduction

The Severe acute respiratory syndrome coronavirus type 2 (SARS-CoV-2) has caused a long-lasting pandemic that has gradually become endemic in 2023 [1]. Elderly people and those with chronic pre-existing conditions are at a higher risk of a severe course of Corona-virus disease (COVID)-19 [2]. A particular risk is present for persons in home care, who are often cared for by relatives and depend on regular visits by their family members. Caregiving relatives are often middle-aged people who are working and/or have children living in their household [3]. Thus, their social contacts cannot be completely reduced, which puts the person cared-for potentially at risk of infection with SARS-CoV-2 [4].

To help cope with the pandemic, research to develop an effective vaccine was accelerated. In December 2020, the first vaccine against SARS-CoV-2 was licensed in Europe and the European vaccination campaign started on 27 December 2020. Especially at the beginning of the vaccination campaign, the availability of vaccines was limited. In Germany, those willing to be vaccinated were divided into prioritisation groups and initially only those over 80 years of age, residents and staff from nursing homes, and people with a particularly high risk of infection were vaccinated. At the beginning of June 2021, vaccine prioritisation was stopped and persons above the age of 12 became eligible for vaccination [5].

However, vaccination does not fully protect against coronavirus infection. One characteristic of SARS-CoV-2 is, that virus variants can develop, which evade the host immune response and reduce the effectiveness of available vaccines. These variants differ in viral kinetics, viral load, risk of transmission, and resulting symptoms. For the wild-type virus, the incubation period averages five days [6–8]. Even before the onset of symptoms, the viral load in the upper respiratory tract rises sharply. Several studies have shown that viral excretion begins one to three days before the first symptoms appear [7–10]. Many patients develop only mild symptoms, and in 20% of infected individuals, symptoms are absent altogether [6, 11, 12], leading to unnoticed viral spread [6, 7].

Testing for SARS-CoV-2 and physical separation after a positive test result can block transmission. The cost-benefit ratio of regular testing of asymptomatic persons is unclear [13, 14]. It may offer an advantage for persons with frequent contacts to a vulnerable person, who may be immunosuppressed or has contraindications for vaccination. Regular asymptomatic testing may also be beneficial, when a new virus variant emerges against which there is no vaccine available, provided that the test detects the new virus variant. However, when new variants appear, the vulnerable population may also change, and the target group for regular asymptomatic testing may need to be redefined.

A test suitable for regular testing should be simple, inexpensive, provide quick results, be portable and durable, and as accurate and reliable as possible [15]. With the introduction of rapid antigen lateral flow assays (point-of-care tests) in autumn 2020 [4], it became possible to obtain a test result quickly and self-tests allowed a location-independent use. While polymerase chain reaction (PCR) tests are the current gold standard, they are expensive, time-consuming, and not ubiquitously available. Lower sensitivity than PCR testing may be acceptable, since self-tests are regarded as one component in infection control alongside social distancing, mouth-nose masks and vaccinations [16].

The willingness to test regularly is due in particular to the altruistic approach of protecting others from infection [16–18]. Betsch et al [19] reported that 72% of survey respondents were willing to test themselves twice a week as part of public health screening in order to quickly identify infectious individuals. In particular, medical staff were willing to participate in testing.

Home care provided by care-giving relatives is an essential part of the care system. However, there are currently no recommendations for testing this group of individuals as there are, for instance, for hospitals carers. We consider that the use of regular asymptomatic testing through a rapid antigen test by care-giving relatives can protect vulnerable individuals in the home care setting.

We conducted a randomised feasibility trial to evaluate if a study investigating different test frequencies is feasible with regard to participant recruitment and retention, as well as the acceptability of regular self-testing. The trial aimed to investigate the participants’ confidence in test performance and results, and their level of concern about infecting themselves and the person cared-for. We also evaluated the vaccination rate and willingness to receive vaccination among care-giving relatives. Additionally we were interested in finding out whether regular asymptomatic self-testing leads to a decrease in direct protective behaviour (mask wearing). This served to inform the design of a subsequent definitive randomised controlled trial to determine whether regular asymptomatic testing through rapid antigen test by care-giving relatives can prevent coronavirus infection of vulnerable people in the home care setting.

## Material and methods

### Study design and setting

In a prospective, parallel-group, randomised feasibility study, we investigated the practicability and acceptance of different test frequencies of a SARS-CoV-2 antigen self-test among care-giving relatives. We conducted the study from 25 March 2021 to 8 July 2021, in the region of Magdeburg, Germany. Participants were randomised 1:1:1 into three groups. Participants in each group performed self-testing for SARS-CoV-2 either once per week (group I), twice per week (group II), or every two days (group III) over a period of 6 weeks.

### Participants

We enrolled adults of at least 18 years who cared for relatives, who still lived in their homes. Caregivers should provide care for at least 30 minutes twice a week. The caregivers were required to have external contacts, for example at work. We placed advertisements in newspapers and on the website of the University Hospital of Magdeburg to recruit participants. The sample size for the pilot study was planned for 45 participants (15 per study arm), based on the expected workload and a minimum of at least 10 patients per arm. Participants were randomised by the study team to one of the three study arms at the first study visit using sealed envelopes. We did not perform block randomisation. We had a total of 45 lots (15 lots per study arm), from which we removed those that were drawn.

### Interventions

At the baseline visit (visit 1), study personnel took a nasopharyngeal swab, saliva samples for PCR, and drew blood for serological measurements to detect an active or previous SARS-CoV-2 infection. Participants completed two questionnaires about their private and work-related contacts, their caregiving activities, the wearing of mouth-nose masks, and their quality of life as well as their attitude towards vaccination. The questionnaires were based on the SeMaCo study [20]. The questions about the practicability and credibility of self-tests were self-developed.

We explained the use of self-administered saliva antigen tests to the participants in detail. Then, participants conducted a test on site under the supervision of the investigator. For the following 6 weeks, participants were advised to self-test and obtain a saliva sample in the morning once a week, twice a week, or every two days, according to study arm. Participants documented the test result by a photograph on their mobile phone, which they presented at the final interview.

In weekly telephone calls (visit 2–7), participants reported their test results to the study staff and answered questions from a follow up questionnaire regarding changes in social contacts and caregiving. In addition, we recorded the fear of transmitting the infection to others, the burden of performing self-tests and whether participants wore a face mask when interacting with the person cared-for. At each visit we reminded participants that regular testing does not prevent infection and recommended wearing a mouth-nose mask.

During the final visit (visit 8), a second blood sample was taken to measure IgG antibody levels against SARS-CoV-2.

The protocol for this trial is available as supporting information (S1 Protocol).

### Ethical approval

The ethics committee of the Otto von Guericke University approved the study (approval number 21/21). The participants received information about the study by mail or E-mail before the first visit. At the first visit, all participants signed the consent form.

### Measurements

According to study arm, participants performed a saliva lateral flow assay and collected saliva for a PCR assay once or twice a week or every two days. We instructed subjects to perform sample collection in the morning before eating or oral hygiene. We qualitatively detected SARS-CoV-2 in saliva samples with a lateral flow immunoassay of the nucleocapsid protein (ulti med^™^ 012G521), according to the manufacturer’s instructions. Briefly, saliva samples were obtained by wiping the tongue and inside of the mouth with a saliva collector until it was saturated with saliva. It was then inserted into the test cassette. Results were read after 10 minutes. Additionally, participants collected saliva for two minutes with the Salivette^®^ system (Sarstedt) and sent it via mail to the laboratory, where we centrifuged the Salivette^®^ to obtain the saliva for PCR testing.

At the baseline visit, we collected nasopharyngeal swabs (eSwab^™^, Copan Diagnostics Inc) for PCR testing. Nasopharyngeal swabs and saliva samples were stored for up to 7 days at 4°C. Both samples were processed identically with established procedures. Briefly, RNA was extracted with the MagNA Pure 96 system (Roche). SARS-CoV-2 was detected by PCR with the LightMix Modular SARS and Wuhan CoV E-gene and the RdRP-gene kit (TIB Molbiol/Roche) in a LightCycler 480II instrument.

For serologic testing, blood samples were centrifuged, serum was collected, and tested for IgG-antibodies against the trimeric spike-protein using the LIAISON^®^ SARS-CoV-2 TrimericS IgG assay (DiaSorin).

Acceptability was defined as adherence to scheduled self-testing and confidence in test results. To evaluate practicability of self-testing we assessed the perceived burden of the test procedure as well as the confidence in performing the self-tests on a scale from 1 (not at all) to 7 (very much). The feasibility outcomes were reported descriptively and narratively. The adherence to the testing regime was calculated with 95% confidence interval (CI). Ordinal data were reported using means and standard deviation, while nominal data were reported using raw counts (number, percentage). Trends over time in the proportion of using mouth-nose masks and mean reported testing burden were evaluated by linear regression. The average change per week (slope) was estimated by treatment group (with p-value and 95% CI). Data from the questionnaire were analyzed with IBM SPSS Statistics 26 (IBM Corp., Armonk, New York) and Excel (Microsoft Corporation). Missing data were not counted. Results are reported following the CONSORT guidelines for Pilot and Feasibility Studies (S1 Checklist).

## Results

**Fig 1:**
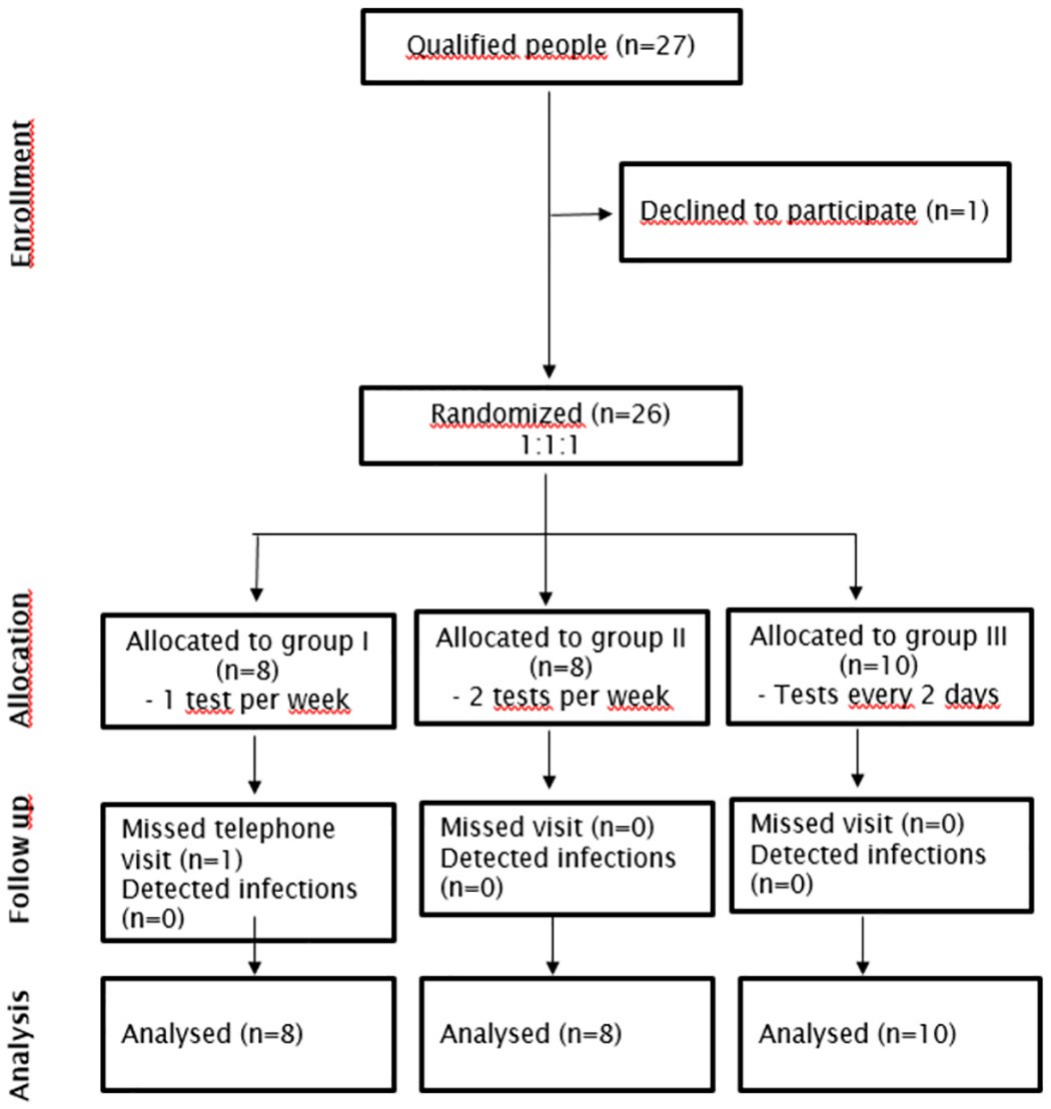
Participant flow diagram.

Twenty-seven participants were assessed of whom 26 were successfully enrolled in the study (Fig 1). The participants were randomised as follows: eight participants in group I (once per week), eight in group II (twice per week) and ten in group III (every second day). All participants completed the study. Baseline characteristics were similar between groups (Table 1). The median age of participants was 54.5 years and 20 (77%) were female. Thirteen (50%) participants worked full time. Nine participants (35%) lived with the person cared-for in one household, and 11 participants (42%) had a high burden of care work with more than 20 hours per week. Participants from group II and III had a higher burden of weekly care work than group I. Participants from group III worked less often in full time (30% vs. 63%). Half of the participants received help from other parties, such as an ambulatory nursing service (13/26, 50%). Of these 12 participants (92%) reported that the nurses wore facemask and protective gear.

**Table 1:** Baseline characteristics of participants. Unless otherwise stated shown as n (%).

	Group I (1 test/week) N = 8	Group II (2 tests/week) N = 8	Group III (testing every two days) N = 10
Age, median (range)	57 (41–63)	52 (30–61)	58 (50–76)
Female	6 (75)	7 (88)	7 (70)
Full-time work	5 (63)	5 (63)	3 (30)
Part-time work, Short—time work, partial retirement, minor employment	2 (25)	1 (12.5)	5 (50)
Non-working	1 (12)	2 (25)	2 (20)
**Housing conditions**			
Number of persons in the household of the caregiver, mean ± standard deviation	2.3 ± 1.4	2.8 ± 0.7	2.3 ± 1.1
Caregiver and person cared-for in different households	6 (75)	4 (50)	7 (70)
**Burden of care**			
>20 hours of care/week	1 (13)	5 (63)	5 (50)
Fear of infecting the person in care	4 (50)	7 (88)	6 (60)
Face mask worn during care work	3 (38)	3 (38)	3 (30)

A total of 378 lateral flow self-tests were performed. At the baseline visit, 28 tests were performed under supervision. Two tests had to be repeated, one due to an invalid and one due to a positive result, the other ones were negative. Of the 350 tests carried out unsupervised at home, 344 were negative, and six (0.02%) invalid. A total of 48/48 self-tests were carried out at home in group I (100.0%; 95% CI 92.6% to 100.0%), 93/96 self-tests in group II (96.9%; 95% CI 91.2% to 98.9%) and 209/210 self-tests in group III (99.5%; 95% CI 97.4% to 99.9%). Of 375 saliva samples collected, 26 were taken during the baseline visit and the remaining 349 at home, of which 344 were received in the lab and tested for SARS-CoV-2 by PCR. Of all 370 samples available in the lab, five could not be evaluated due to insufficient amount of saliva or inconclusive test results. The remaining 365 saliva samples were PCR-negative.

**Fig 2:**
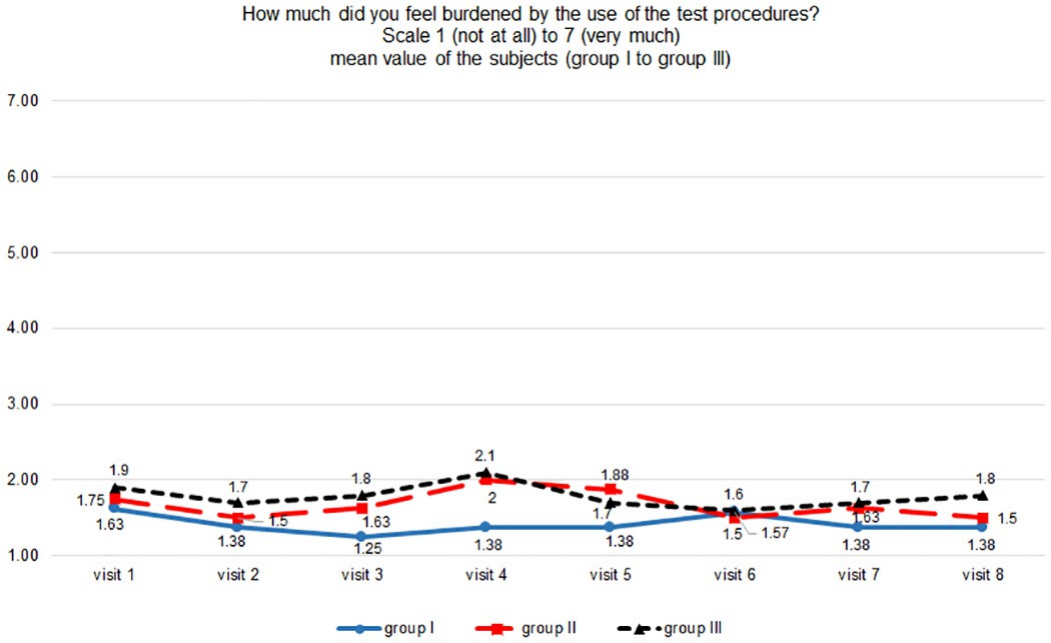
Perceived burden of the test procedure (as assessed by the questionnaire, scale 1 to 7).

Participants did not perceive regular self-tests as stressful (Fig 2). There were no differences in reported testing burden, i.e. group I -0.01 (95% CI -0.06 to 0.04) units, group II -0.02 (95% CI -0.07 to 0.03) units, group III -0.02 (95% CI -0.07 to 0.03) units.

**Fig 3:**
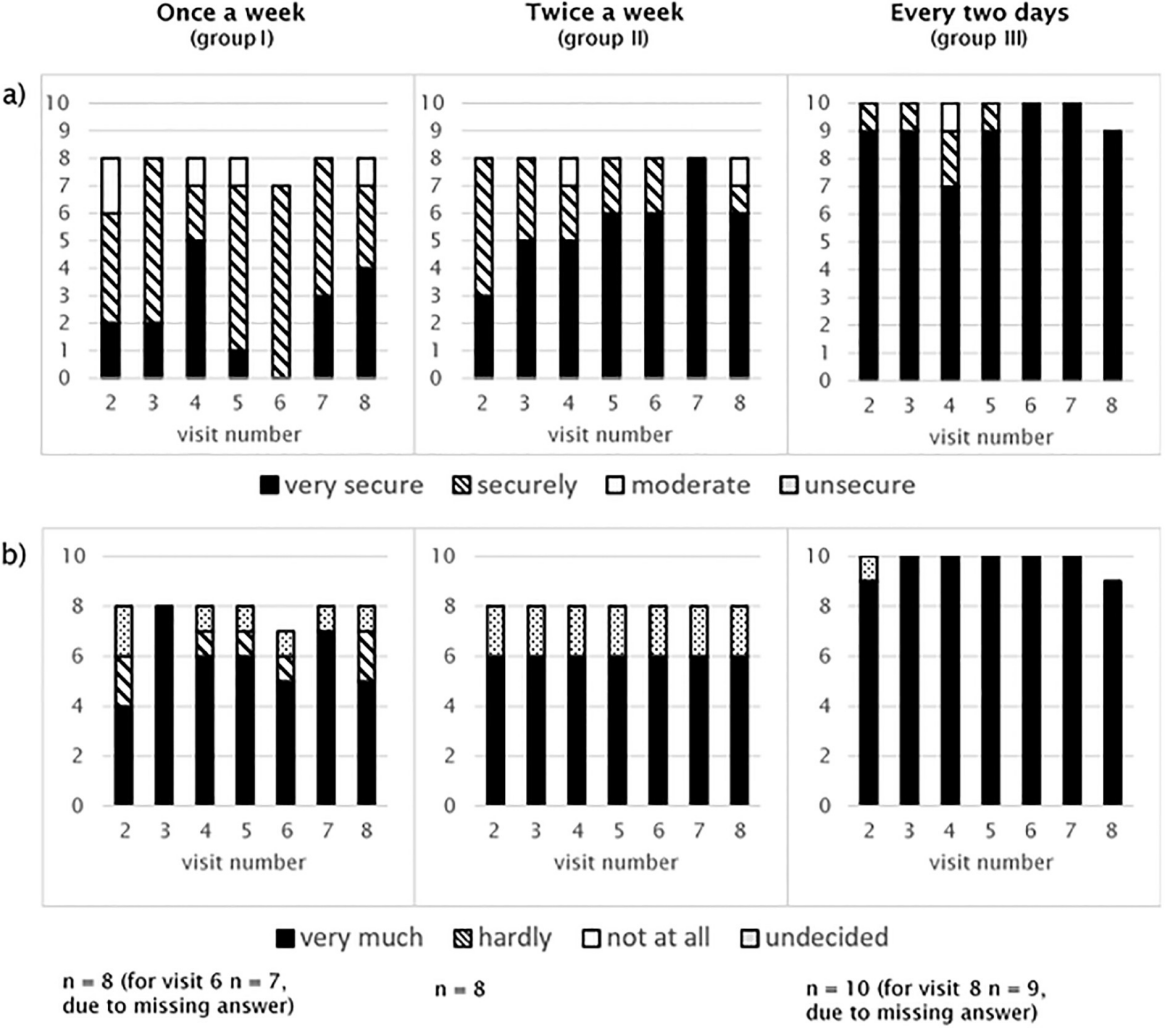
(a) Confidence in performing the saliva self-test. (b) Trust in the result of the saliva self-test.

Participants in group III with a high frequency of testing reported higher confidence in performing self-tests than group I. The confidence in the test result was greater with increasing frequency of testing (Fig 3).

The majority (17, 65%) of the study participants was afraid of infecting the patient with SARS-CoV-2. On a scale from 1 (not at all) to 7 (very much), participants had moderate fear of infecting themselves with the new coronavirus or infecting someone else (average 3.19±2.17 and 3.62±2.43 at baseline, respectively). This did not change during the study period (average 3.19±2.26 and 3.27±2.39 at the final visit, respectively). There was no apparent change within and between the individual groups over the course of the study.

**Fig 4:**
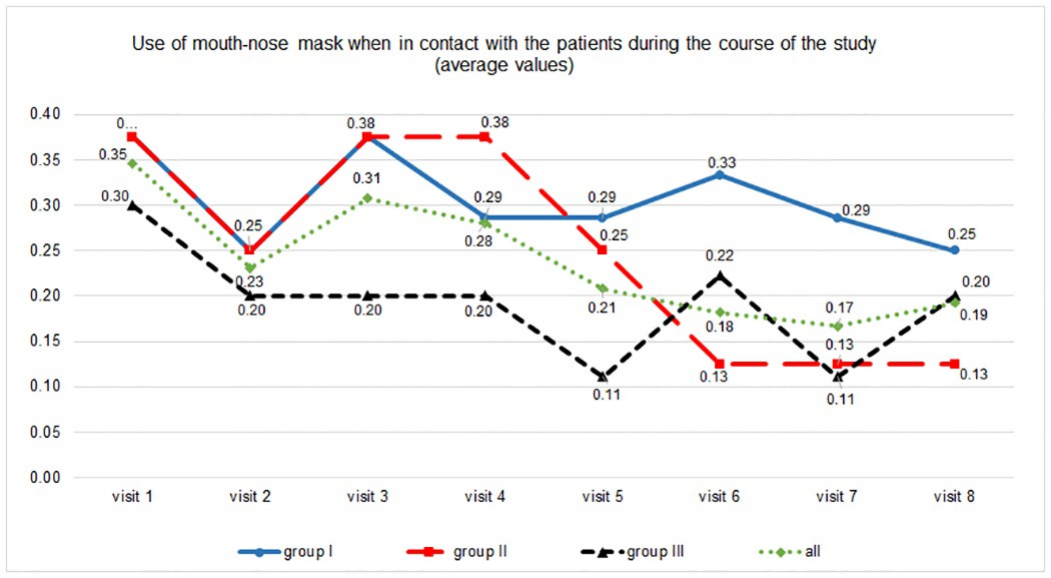
Use of mouth-nose masks during care over the course of the study.

Nine participants (35%) reported wearing mouth-nose masks during care. Adherence to wearing the mouth-nose masks during care decreased to 19% over the course of the study (Fig 4). Groups differed in their decline (rate per week) of wearing masks, i.e. group I -2.0 (95% CI -3.9 to 0.0) % with p = 0.051, group II -4.1 (95% CI -6.1 to -2.1) % with p<0.001, and group III -1.4 (95% CI -3.4 to 0.6) % with p = 0.156, p value for interaction group*time <0.001. Of those who lived in the same household as the person being cared for, only one person reported wearing a mouth-nose mask during care work (Table 2).

**Table 2:** Contingency table of mask wearing and the site of care at baseline visit.

		**Do you wear a face mask?**	
		Yes	No
**Care in the common household**	Yes	1	9
	No	8	8

At the beginning of the study, about one-third of participants (9 of 26) had received one dose of a vaccine against COVID-19. One study participant was unable to receive the vaccine due to a previous PCR-positive SARS-CoV-2 infection. Of the 17 unvaccinated participants, 13 (77%) reported that they planned to be vaccinated. At the end of the study, 18 participants (69%) had received a vaccine. When participants were asked whether vaccination should be mandatory, 10 (39%) favored it, nine (35%) opposed it, and seven (27%) were undecided. Antibodies against SARS-CoV-2 were found in the serum of 18 vaccinated or recovered individuals. One vaccinated participant probably had not yet developed antibodies because vaccination had occurred only recently. No further participant developed antibodies against SARS-CoV-2 during the study period.

## Discussion

In a randomized pilot study, we assessed the feasibility of three frequencies for regular asymptomatic testing in caregiving relatives. We showed that conducting such a study is feasible and that saliva-based antigen self-testing every two days was well tolerated. Over time, participants grew more comfortable with performing the test procedure. However, mask adherence during care decreased in all groups.

The setting of non-professional, care-giving relatives is unique because there is a strong relationship between caregiver and person in care. Of note, the majority of caregivers was afraid of infecting the persons they cared for, and they showed high compliance in performing self-tests. The high level of motivation to comply with the requirements of the study was remarkable. We speculate that the study selected for participants who considered regular self-tests to be helpful for protecting the person being cared for, and that care-giving relatives show high compliance because they feel altruistic about protecting the person they care for. Therefore, our results are not applicable to the general public.

The course of the pandemic may have played a role in motivating participants to conduct regular asymptomatic self-testing. At the start of the study in late March 2021, the 7-day incidence rate of confirmed SARS-CoV-2 infections in Germany was 123 cases per 100,000 inhabitants with a case fatality rate of 1,197 deaths per week [21]. At this point in time, 4.3% of the total population had received basic immunisation with two vaccine doses [22]. The initially predominant wild-type virus particularly had affected older people and those with pre-existing conditions. With the emergence of N501Y-positive variants of concern from April 2021 onwards, the hospitalisation rate rose from 4.3% to 5.4%, with an increase of infection and hospitalisation rates in the younger population [23]. Thus, the actual and perceived risk of harm may have influenced the willingness to self-test, which is why the study results may not be transferable to other pandemic phases. Should a more harmful variant of the virus emerge for which there is not yet an adapted vaccine, regular asymptomatic self-testing may regain importance for members of risk groups and their caring relatives.

In other studies, participants preferred at-home testing, rather than community based testing sites [24, 25]. Our study participants reported that they tolerated at-home testing very well and integrated it into their daily routine. We chose saliva tests because they are less invasive and, therefore, better tolerated than nasal swabs [26], which have also become increasingly available from mid-March 2021 [27]. In addition, we did not want participants to handle liquids, as is the case with lateral-flow tests using nasal swabs. At the time the study was planned, there were hardly any studies comparing the different types of rapid tests. Recent studies showed that saliva tests and buccal swabs are inferior to nasopharyngeal swabs or swabs from the anterior nose [26, 28].

Participants reported increased confidence with the testing procedure over time and we found a correlation between the number of tests performed and perceived trust in the test results. As an unexpected side effect, participants reported reduced mask wearing during the course of the study (from 35% to 19%), despite recurrent reminders of the limited sensitivity of the tests, with the strongest decline in the group with twice weekly testing.

It is controversially discussed in the literature, whether frequent testing influences the adherence to public health measures. For example, a survey by Betsch et al. [19] found that subjects were less likely to wear masks and keep distances after a negative test result. However, if the study participants were made aware of limited test performance, this tendency would be reduced. On the other hand, a survey by Wanat et al. [16] and Christensen et al. [18] found that a negative self-test does not influence adherence to public health measures.

In the first two months of the study period, contact restrictions were instituted in Germany due to a high incidence of infection and rising hospitalisation rates. For the region in which the study was conducted, Saxony-Anhalt, this meant that a maximum of five people from a maximum of two households were allowed to meet, public events and tourist trips were banned, facilities such as theatres, sports facilities, and meeting places for the elderly were closed, the number of customers in shops was restricted, and restaurants were only allowed to offer take-out food [29]. From mid-June 2021 when the incidence fell and vaccination rates increased, restrictions were gradually lifted from, especially for vaccinated, recovered, and tested people [30]. The relaxation of restrictions may have contributed to the fact that participants wore less mouth-nose masks later in the study.

We observed that invalid test results could be unsettling to participants. Thus, it is important to provide a point of contact for participants to discuss invalid results. Positive results should be validated promptly by PCR testing to minimise possible confusion and unnecessary isolation measures. In a study by Hoehl et al, where teachers in Germany performed a SARS-CoV-2 self-test, 76.2% of positive self-tests were identified as false positives by subsequent PCR [31]. Especially in populations with a low prevalence of SARS-CoV-2 infections, false positive results can be a problem [32]. It could be possible that a person may ignore a subsequent true positive or stop regular asymptomatic self-testing, if they previously had a false positive result.

It is worth noting that one third of the study participants were vaccinated, at a time when it was still very difficult in Germany to get a vaccination appointment. Initially, 13 (76.5%) of the 17 unvaccinated participants wanted to be vaccinated, and we documented vaccination of nine participants during the study period. This demonstrates the high willingness to be vaccinated among care-giving relatives.

We performed PCR testing on saliva samples to ensure no infections were missed during the study period. Furthermore, antibodies were only detectable in participants who reported either a previous vaccination or infection. It is important to note that only anti-spike antibodies were measured, and not anti-nucleocapsid antibodies. This means that serological differentiation between infection and vaccination is not possible.

This feasibility study has several limitations. First, we could not enroll the planned sample size during our recruitment period. This may be due to the fact that a large-scale vaccination program was under way, which may have distracted attention from self-testing. Furthermore, non-mandatory testing was implemented in businesses and public testing centers [33], so the perceived need for additional self-tests in the population may have been low. Care-giving relatives often have a busy time schedule due to the burden of work and caregiving, and may not have found time to participate in the study. Second, we chose simple questionnaires to assess fear of infection rather than standardised test instruments, which reduces comparability of the results with other studies. However, we felt that participants may not have tolerated longer questionnaires. Third, we did not observe SARS-CoV-2 infections due to the small sample size, short sampling time, the high proportion of vaccinated individuals, and the restriction of social contacts in place. Thus, we could not measure test performance of the saliva tests or whether regular asymptomatic saliva testing could prevent transmission of the virus.

We identified important aspects that need to be considered when conducting a definitive trial. First, the definitive trial should be conducted at a time when there is a high prevalence of infection. Second, characteristics of the viral variant such as the length of the incubation period or the proportion of asymptomatic cases, need to be taken into account, when selecting testing frequencies. Third, active recruitment and multi-centre implementation may be advisable to reach the target group and gain the required number of test subjects. Furthermore, a definitive study should investigate whether regular self-testing leads to a reduction in the use of mouth-nose masks.

In summary, we found that a study assessing different frequencies of self-testing is feasible. Larger studies are needed to assess the optimal frequency of regular asymptomatic self-testing to prevent coronavirus infection of vulnerable people in the home care setting and whether self-testing leads to a reduced use of protective measures.

## Supporting information

S1 Protocol (PDF)

S2 Protocol (PDF)

S1 Checklist (PDF)

S1 Dataset (XLSX)
