# Supplementary material for: Determining the optimal frequency of SARS-CoV-2 regular asymptomatic testing: A randomized feasibility trial in a home care setting
Source: PLoS One. 2024 Jul 3;19(7):e0303344. doi: 10.1371/journal.pone.0303344 (PMC11221670; doi:10.1371/journal.pone.0303344)
Supplement: S1 Protocol — (PDF) [file pone.0303344.s001.pdf]

Study Protocol

SuRIP/B-FAST

SARS-CoV-2

Risk-based surveillance of persons with close contact to persons from risk groups in the context of home care in the Magdeburg area in the SARS-CoV-2 pandemic

Acronym: SuRIP

A prospective, longitudinal, regional cohort study to develop surveillance concepts and test their acceptance among caregivers/relatives in the home setting as part of the nationwide research network on applied surveillance and testing (B-FAST).

SuRIP/ B-FAST

Study Protocol Version 1.0

15.02.2021

Study management

Prof. Dr. med. Achim Kaasch
Institute for Medical Microbiology and Hospital Hygiene (IMMB)
Medical Faculty, Otto von Guericke University Magdeburg
Leipziger Str. 44, 39120 Magdeburg
T +49 391 / 67 - 13392, F +49 391 / 67 - 13384
achim.kaasch@med.ovgu.de

Study team

Institute for Medical Microbiology and Hospital Hygiene (IMMB)

PD Dr. med. habil. Ina Tammer
Milica Dilas
Annett Hellriegel-Nehrkorn

Institute of Social Medicine and Health Systems Research (ISMG)

Prof. Dr. Christian Apfelbacher, PhD
Robert Pohl, M.A.
Medical Faculty, Otto von Guericke University Magdeburg

Modelling: Max Planck Institute for Dynamics of Complex Technical Systems Magdeburg

Prof. Dr. Peter Benner
Dr. Sara Grundel
Dr. Christian Himpe
Sandtorstr. 1
39106 Magdeburg

Summary

**Title**	Surveillance of family carers with close contact to persons from risk groups in the Magdeburg area in the SARS-CoV-2 pandemic<br><br>Acronym: SuRIP - SARS-CoV-2<br><br>A prospective, longitudinal, regional cohort study to develop surveillance concepts and to test their acceptance among carers/relatives within the framework of the nationwide research network on applied surveillance and testing (B-FAST).
**Study population**	Relatives caring for persons from risk groups (vulnerable groups), such as senior citizens or persons with disabilities
**Primary objective**	Practicability and acceptance of different test frequencies of a simple, self-performable and self-evaluable test system (saliva test) among caring relatives.
**Secondary objective**	Obtaining data for modelling the test frequency.<br><br>Verification of the test quality (sensitivity and specificity) of self-test systems.<br><br>Investigate the influence of external contacts of the carers (e.g. occupational) and the current incidence of infection and the associated potential risks of transmission of SARS-CoV-2 to the person being cared for.<br><br>Measurement of vaccination acceptance and willingness to vaccinate in this group of people.

**Study design**	Prospective, longitudinal pilot cohort study with serial self-testing using a saliva test initially over a period of 6 weeks. 45 family caregivers are randomised into 3 groups with different test frequencies.<br>1st group: 1 time per week<br>2nd group: 2 times per week<br>3rd group: every 2 days<br>15 people will be randomly assigned to each group (lottery).<br><br>Recruitment: Subjects are recruited by advertisement in the local print media. Interested persons should contact the study secretariat by telephone.
**Investigation scheme**	Visit 0: Selection of subjects according to the defined inclusion and exclusion criteria (minimum age, caring relatives, external contacts, written consents, no active COVID-19 disease; see points 2.3. and 2.4.) at first telephone contact. Randomisation of the selected subjects.<br><br>Visit 1: Personal visit of the subjects to the study centre, collection of a combined nasopharyngeal swab for PCR testing (exclusion of SARS-CoV-2 carrier status), blood collection for antibody determination, completion of the "first contact" and "vaccination" questionnaires by the study participants accompanied by the study staff, instructions for self-testing and documentation.<br><br>Visit 2-7: Weekly telephone contact (individual interviews) to query the test results, query of the "Follow up" questionnaire on the type and frequency of contacts in the last week as well as on changes in the professional and private environment, regular sending in of the salivettes for PCR testing.<br><br>Visit 8: Final visit, personal visit of the study participants, blood sample for antibody determination, questionnaire "final discussion".
**Participating centre**	University Hospital Magdeburg
**Funding**	Funding by the Federal Ministry of Education and Research as part of the B-FAST project of the Nationales Forschungswerk der Universitätsmedizin on Covid-19.

1 Introduction

At the end of 2019, a new coronavirus SARS-CoV-2 (severe acute respiratory syndrome coronavirus type 2) appeared for the first time in China as a causative agent of pneumonia. In January 2020, the virus was identified by the Chinese authorities and reported to the WHO. Since then, the new coronavirus has been spreading worldwide. In March 2020, the outbreak was officially declared a pandemic by the WHO Secretary-General (1).

Coronaviruses cause mainly mild colds in humans, but can also cause severe pneumonia. SARS-CoV-2 causes the disease COVID-19. COVID-19 can manifest itself in many ways and not only in the lungs, but also in other organ systems, such as the nervous system, the gastrointestinal tract, the cardiovascular system or in the kidneys. In addition, a relatively wide range of dermatological manifestations has been described, but these are rare overall (0.2-1.2%). Some patients with severe SARS-CoV-2 infection develop a hyperinflammatory syndrome 8-15 days after the onset of the disease, which can lead to multiple organ failure and is associated with high mortality. Severe courses may also occur in persons without known previous disease and in younger patients. However, severe courses of the disease are observed much more frequently in certain groups of people. These so-called risk groups or vulnerable groups include above all older persons. 86% of those who died of COVID-19 in Germany were 70 years old or older (median age: 82 years) (2).

The proportion of people in need of care increases with age. According to the Federal Statistical Office, 4.13 million people in Germany were in need of care within the meaning of the Long-Term Care Insurance Act (SGB XI) at the end of 2019. Four out of five people in need of long-term care (80% or 3.31 million) were cared for at home. Of these, 2.33 million persons in need of long-term care were cared for mainly by relatives (3). As a rule, the family caregivers not only have close contact with the person to be cared for, but also frequently have other external contacts, for example through their professional activities, which poses a potential risk of infection and transmission to the person to be cared for.

In addition, new SARS-CoV-2 variants are also spreading in Germany, for which the effects cannot yet be assessed. Since mid-December 2020, the United Kingdom (UK) has been reporting the increasing spread of the SARS-CoV-2 lineage B.1.1.7 (VOC 202012/01; VOC: variant of concern), which is characterised by an unusually high number of mutations, particularly in the viral spike (S) protein, with the help of which the virus attaches to human cells. It is now assumed that this variant exhibits increased transmissibility, resulting in a higher number of replications (2).

Herd immunity to protect against COVID-19 can be achieved either through a high vaccination rate or through natural infection. Herd immunity through natural infection would require a large population group (more than half) to undergo the disease and produce antibodies. However, it is not yet clear whether everyone acquires full immunity after passing through infection and how long this lasts. This scenario would be associated with a high number of seriously ill patients and deaths and would not be ethically justifiable (4).

The only currently viable way to achieve sufficient herd immunity is vaccination. Currently, 3 vaccines are licensed in Germany. However, due to the high demand, these cannot yet be provided in sufficient quantities. According to the RKI, only 1.5% of the adult population in Saxony-Anhalt is currently fully immunised (2 vaccinations) (5). To make matters worse, according to the recommendation of the STIKO (Standing Committee on Vaccination at the Robert Koch Institute), the vector-based COVID-19 vaccine from AstraZeneca is currently only recommended for people aged 18 to 64 years, based on the data currently available. For these reasons, the RKI has defined priority risk groups to be vaccinated who have a particularly high vulnerability or exposure risk (level 1=highest priority). Since most family caregivers are most likely to be assigned to level 3 or a higher level in the STIKO's prioritisation list, they will only benefit from this protective measure with a time lag (6).

Important measures to contain the pandemic are adherence to the AHA-L rules (distance-handwashing-everyday-mask-ventilation), quarantine measures in case of infection or contact

with an infected person and testing in case of suspected infection (7=testing criteria). PCR from nasopharyngeal swabs, throat swabs or combined nasopharyngeal swabs is still considered the gold standard for testing. The use of other sample materials, such as pharyngeal rinse water/ gargle water and saliva, is being discussed, as the collection of these materials is significantly better tolerated. However, there are significantly fewer empirical values for these materials. For saliva, some groups describe a lower clinical diagnostic sensitivity (8, 9), while other groups found comparable or, in the case of some studies, even higher sensitivity of PCR diagnostics compared to bilateral nasopharyngeal swabs (10, 11).

Antigen tests are increasingly used to relieve the burden of PCR diagnostics. The RKI's "National Test Strategy SARS-CoV-2" recommends that staff e.g. in care facilities and in outpatient care without a COVID-19 case in areas with an increased 7-day incidence (>50/100,000 inhabitants) should be regularly tested e.g. by means of antigen testing. For regular serial testing, the test regulation provides for an entitlement to testing once a week (12,13).

Due to a lack of studies and a possibly lower sensitivity, screening of asymptomatic individuals is currently discouraged, as test results only provide a snapshot. However, modelling and initial population-based studies suggest that the most important factors for effective surveillance are the frequency of testing and the speed with which a result is available, rather than the sensitivity of the testing system (14, 15 ,16).

1.1 Need for a study

Although family caregivers have a very high share in the care of vulnerable persons and therefore the protection of this group is also very important, there are no recommendations for testing this group of persons so far. It is also unclear how high the proportion of people with a previous infection currently is, how many people have already been vaccinated and what the general attitude towards vaccination is in this population group.

The collection of these data is necessary in order to develop effective surveillance concepts for this population group, which in turn increase the protection and safety of the group of people to be cared for.

1.2 Objectives

The **primary objective** of the study is to determine the practicability and acceptance of different test frequencies of a simple, self-performable and self-evaluable test system (saliva test) in the previously underrepresented population group of family caregivers.

Important **secondary objectives** are:

1. obtaining data for modelling test frequency, socio-demographic data for improving mathematical models and for developing realistic surveillance systems.
2. to test the test performance (sensitivity and specificity) of self-testing systems.
3. to investigate the influence of external contacts of the carers (e.g. occupational) and the current incidence of infection and the associated potential risks of transmission of SARS-CoV-2 to the carer.
4. measure vaccination acceptance and willingness to vaccinate in this group of people.

2 Methods

2.1 Design pilot study

Prospective, longitudinal cohort study with serial self-testing with a saliva test (self-performable rapid antigen test from saliva samples) as a pilot phase over a period of 6 weeks on 45 persons. The following frequencies of self-testing will be investigated:

Group 1: 1 time per week

Group 2: 2 times per week

Group 3: every 2 days

Fifteen people are randomly assigned to each group. The allocation will be made via lots in prefabricated envelopes, which will be opened during the first visit.

The pilot phase with 45 people is to start in March 2021 (planned study period 15.03.2021 to 30.04.2021).

At the first contact (visit 1), the study design will be explained in detail to the test persons in a personal interview. They will also be informed about the blood collection and the taking of a nasopharyngeal swab for PCR testing.

After the subjects have given their written consent, the study doctor will take a blood sample to detect the presence of antibodies against SARS-CoV-2 as an indication of a previous infection and a combined throat and nasal swab to determine the SARS-CoV-2 carrier status (infection). The results will be communicated to the test persons promptly by telephone and in writing.

During this visit, the volunteers will be instructed on how to perform the saliva test, which they will carry out once on their own on site. Subsequently, the evaluation of the test is explained to the test persons. In addition, they are instructed on how to perform the saliva collection with the Salivette® system. They also carry out this test once independently on site.

In addition to the respective tests, the subjects will be given two questionnaires (questionnaire "First contact" and "Vaccinations"), which they are to complete together with the study staff at the study centre.

At the end of the first interview, each subject will be given the following materials and documents:

- corresponding number of saliva tests (depending on the randomisation group)
- appropriate number of salivettes
- written instructions for the saliva test
- form for documenting the test results (forms for documenting self-test results, Appendix 7.4.)
- stamped envelopes for the return of the salivettes
- a copy of the consent form for participation (Appendix 7.2)
- Information sheet on participation in the study (participant information, Appendix 7.1)

In the following weeks, the test persons should carry out the quick test alone at home and enter the test result in a documentation sheet. In addition, the volunteers are asked to take photos of the test cartridges with their mobile phones if possible and to bring the photos to the final interview. Furthermore, the study participants should collect saliva with the salivette system after performing the saliva test and send the sample tube to the study centre by post.

The test persons are contacted once a week by telephone by the study centre (individual interviews) in order to record possible changes from the initial interview (questions on personal contacts, questions on changes in the professional and private environment, etc.) and current surveys on the acceptance and practicability of the rapid tests and salivette systems on a weekly basis with the help of a structured questionnaire (questionnaire "Follow up"). At the same time, the result of the rapid tests is requested and an appointment is made for the next telephone survey.

At the end of the examination interval, a personal final interview takes place in the study centre, in which data on acceptance and practicability as well as suggestions for improvement on the part of the test subjects are collected again with the help of a structured questionnaire ("final interview" questionnaire). A second blood sample will be taken for a new antibody test in order to record recent infections.

Recruitment of participants

The subjects are recruited by advertisement in the local print media. Interested persons should contact the study secretariat by telephone.

Follow-up recruitment

If subjects leave the study during the first week, a follow-up recruitment will take place. For this purpose, interested persons who responded to the advertisement but could not be included in the initial recruitment will be contacted by telephone.

2.2 Schedule

Milestones:

19.02.2021<br>Ethics submission	15.03.2021<br>Start pilot phase (6 weeks)	15.06.2021<br>End of data collection	01.08.2021<br>End of data evaluation

Pilot study

	Jan	Feb	Mar	Apr	May	Jun	Jul	Aug	Sep
Preparation	■	■	■						
Recruitment			■						
Study inclusion			■	■					
Study period				■	■				
Study finalisation						■	■		

2.3 Inclusion criteria

- Minimum age of 18 years
- Family carers with at least 2 contacts per week of at least 30 minutes duration with the person to be cared for.
- Caring relatives have external contacts (e.g. occupation)
- Written informed consent to participate in the study.
- Written consent for blood sampling (2x) and for taking a nasopharyngeal swab (1x).

2.4 Exclusion criteria

- active COVID-19 disease

2.5 Procedure in case of withdrawal of consent

If a subject withdraws his/her consent to participate in the study, this must be done in writing. The subject will then receive written confirmation of receipt. The data collected up to that point will be analysed.

2.6 Procedure in case of occurence of COVID-19 infection during the study

The test persons are informed that if symptoms of a cold appear, they must immediately take measures to distance themselves and contact their family doctor. The general practitioner decides on the clarification of an acute COVID-19 disease. Alternatively, the test persons will be referred to the fever outpatient department of the university hospital for testing by means of PCR. The volunteers are informed that the self-test does not replace these measures.

If a COVID-19 infection is detected, the volunteers must inform the study centre. They will then be excluded from the study.

2.7 Procedure in case of positive saliva test

If the saliva test is positive, the subject must immediately contact the study centre by telephone.

To exclude a COVID-19 infection, a combined throat and nose swab will be performed by trained staff at the study centre or at the fever outpatient clinic at Magdeburg University Hospital and a PCR test for SARS-CoV-2 will be performed.

If the PCR result is negative, an infection is excluded at the time of sample collection. The test person will receive a self-monitoring form to be completed daily and will initially remain in the study. If symptoms of a COVID-19 infection occur, the study centre must be informed and the family doctor contacted (see point 2.6).

If the PCR result is positive, a SARS-CoV-2 infection is confirmed. The subject must then go into home isolation. The result of the PCR test is communicated to the public health department, which will arrange for further measures.

2.8 Procedure in the event oft he death oft he person being cared for

If the person being cared for dies during the course of the study, the participant no longer belongs to the group of people being studied and is excluded from the study. The test person may keep the saliva tests that have been handed out and not yet used up to this point.

2.9 Data collection and data flow

Data collection:

There will be 2 visits with personal presentation at the study centre. The other contacts are made by telephone in the form of individual interviews.

Visit schedule:

	Visit 1 Inclusion	Visit 2 Week 1	Visit 3 Week 2	Visit 4 Week 3	Visit 5 Week 4	Visit 6 Week 5	Visit 7 Week 6	Visit 8 Closing
Personal visit	×							×
Antibody determination Blood sample 10ml serum	×							×
PCR test/ nasopharyngeal swab	×							
Questionnaire	×							×
Randomisation	×							
Self-test by participants (saliva test, salivettes)	×	×	×	×	×	×	×	
Query test result (by telephone)		×	×	×	×	×	×	
Questionnaire (by telephone)		×	×	×	×	×	×	

The initial survey, vaccination acceptance and willingness to be vaccinated are recorded pseudonymously with paper questionnaires during the initial interview at the study centre and are kept by the IMMB during the pilot phase and forwarded to the Institute for Social Medicine and Health System Research (ISMG) of the University Medical Centre Magdeburg for later evaluation. The telephone survey of the follow-up questionnaires is conducted by IMMB staff and is also passed on to the ISMG in pseudonymised form after completion of the pilot study.

Data storage

Pseudonymised blood analysis results: Laboratory EDP system of Magdeburg University Medical Centre.

Pseudonymised survey results: Survey data are stored from questionnaires (both telephone and paper) in the ISMG. The data storage is pseudonymised.

Randomisation and pseudonymisation

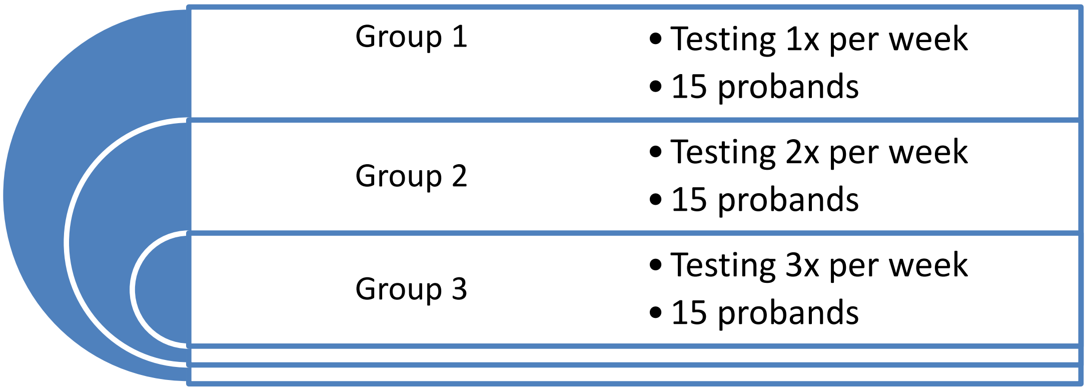

Fig.1

In a lottery procedure, the test persons draw a neutral envelope containing the participant number and thus the respective group assignment (Fig. 1).

Data flow

A systematic overview of data collection and data flow is given in the appendix under 7.5.

The questionnaires completed by the test persons are given a participant pseudonym (TN-ID) by the IMMB. The same is done by the interviewer during the telephone interview of the follow-up questionnaires. This TN-ID remains the same for each participant during the pilot study.

The antibody status (test results) is extracted from the antibody results and stored separately together with the TN-ID.

The results of the PCR test, antibody test and saliva test are transmitted to the ISMG with the corresponding TN ID and stored there until the end of the study.

In the ISMG, the survey data are linked together with the data on the tests via the TN-ID. The data set is stored as an evaluation data set.

The analysis of the evaluation data set by the researchers of the study follows.

2.10 Laboratory tests

2.10.1 Sample collection

At the first contact, a blood sample of 10ml (serum monovette) is taken by peripheral venipuncture and a mouth-nose swab for RT-qPCR is taken in the outpatient clinic of the IMMB (Personal Screening Outpatient Clinic).

The subjects will perform the first saliva test at the study centre under supervision. The COVID-19 Antigen Saliva Test Card, ultimed Products Deutschland GmbH, Ahrensburg, Germany, will be used for this purpose. The sufficient availability of the test kits will be checked before the start of the study.

The other saliva tests will be performed independently at home. The reading of the test result is done independently by the study participants. The test results are recorded in a document and photographed if possible.

Parallel to the saliva test, the study participants collect saliva with the Salivette system (Salivette®, Sarstedt, Nümbrecht, Germany). The sample tube is sent to the study centre in a stamped envelope.

The samples are recorded via the laboratory EDP system of the IMMB with the study number (pseudonym) and stored for 30 years.

Serological and molecular biological tests

The LIAISON SARS-CoV-2 TrimericS IgG assay (DiaSorin) is used to determine antibodies against SARS-CoV-2. If the antibody result is positive, a test with the "ImmuSAFETM Covid+" system (Aicone) is carried out to distinguish antibodies against endemic coronaviruses.

The determination of SARS-CoV-2-RNA is carried out using commercial test systems in the diagnostic area of the Institute of Medical Microbiology and Hospital Hygiene.

2.10.2 Specimen storage

All samples are stored at -80°C in the IMMB for 2 years.

2.11 Ethical considerations

2.11.1 Consent

A written declaration of consent is provided (Appendix 7.2.). This refers to the objective and content of the study, the measuring instruments, the type and content of data collection and storage as well as the voluntary nature of participation and the possibility of revoking consent at any time. Participants will not suffer any disadvantages if they do not participate. Participants will be given sufficient time to consider their decision before giving their consent/refusal. If you have any questions, please contact the staff of the Institute of Medical Microbiology and Hospital Hygiene.

2.11.2 Risks and benefits

Blood is drawn by medical staff at first contact. There is no significant risk of infection or injury during diagnostic blood sampling. Psychologically induced circulatory problems may occur, in very rare cases shock. Relatively often, however, a bruise develops at the puncture site. In rare cases, a pharyngeal swab can cause an injury to the mucous membrane or trigger a nausea. There are neither direct advantages nor disadvantages for the test persons. They have access to self-testing (test kits provided free of charge) and antibody testing.

2.11.3 Data protection

Data processing (collection, storage, use) within the scope of the SuRIP study is carried out on the basis of the data protection concepts of the University Medical Center Magdeburg. Data collection in the context of the survey (by telephone) is password-protected on the server of the IMMB). The data protection concepts conform to the EU Data Protection Regulation (EU-DSGVO) and the Federal Data Protection Act.

Data storage locations:

Person-identifying data: Patient information system of the IMMB, password-protected.

Pseudonymised survey results: permanently in the IMMB

Data access:

Data access to the study data is only possible for the IMMB staff involved in the study. A list of names with the participant identification number (TN-ID) is stored at the IMMB under password protection.

Linking with personal identifying data from the patient information system via the ID-TN is excluded.

Pseudonymisation:
In a lottery procedure, the test persons draw a neutral envelope containing the participant number and thus the respective group assignment.

2.11.4 Staff Savety

Protection against COVID-19 infections is provided in accordance with the applicable hygiene guidelines of UMMD.

2.12 Funding

Funded within the framework of the "Federal Research Network Applied Surveillance and Testing".

3 Statistical Analysis

Descriptive evaluations are planned for the statistical analysis of the data.

3.1 Epidemiological indicators

Within the group of family caregivers, frequencies of SARS-COV 19 infection and surveillance are investigated and differentiated according to sociodemographic characteristics and contact frequencies.

4 Communication of the results

The results are to be made freely accessible via scientific publications in international and national journals with peer-review procedures in open access.

If applicable, results will be communicated through lectures and posters at national and international congresses, as well as through presentations at events.

5 Literatur

1. https://www.euro.who.int/de/health-topics/health-emergencies/coronavirus-covid-19/novel-coronavirus-2019-ncov. WHO2021 abgerufen 09.02.2021

2. Robert Koch Institut. Epidemiologischer Steckbrief zu SARS-CoV-2 und COVID-19. https://www.rki.de/DE/Content/InfAZ/N/Neuartiges_Coronavirus/Steckbrief.html;jsessionid=A4CD5057E0CB43880DA27789570EC7D1.internet052?nn=2386228, download 2021-02-11)

3. Statistisches Bundesamt (Destatis). Pflegestatistik 2019. Deutschlandergebnisse. https://www.destatis.de/DE/Themen/Gesellschaft-Umwelt/Gesundheit/Pflege/_inhalt.html, download 2021 02 11.

4. Papachristodoulou E, Kakoullis L, Parperis K, Panos G. Long-term and herd immunity against SARS-CoV-2: implications from current and past knowledge. Pathog Dis. 2020;78(3):ftaa025. doi:10.1093/femspd/ftaa025.

5. Robert Koch Institut. Digitales Impfquotenmonitoring zur COVID-19-Impfung. https://www.rki.de/DE/Content/InfAZ/N/Neuartiges_Coronavirus/Daten/Impfquoten-Tab.%20html. download 2021-02-11.
6. STIKO-Empfehlung zur COVID-19-Impfung, Epidemiologisches Bulletin 5 | 2021 4. Februar 2021
7. Robert Koch Institut. Testkriterien für die SARS-CoV-2 Diagnostik bei symptomatischen Patienten mit Verdacht auf COVID-19. https://www.rki.de/DE/Content/InfAZ/N/Neuartiges_Coronavirus/Teststrategie/Testkriterien_Herbst_Winter.html. Download 2021-02-21.

8. Chen, J.H., Yip, C.C., Poon, R.W., Chan, K.H., Cheng, V.C., Hung, I.F., Chan, J.F., Yuen, K.Y., and To, K.K. (2020). Evaluating the use of posterior oropharyngeal saliva in a point-of-care assay for the detection of SARS-CoV-2. Emerg Microbes Infect *9*, 1356-1359.
9. Jamal, A.J., Mozafarihashjin, M., Coomes, E., Powis, J., Li, A.X., Paterson, A., Anceva-Sami, S., Barati, S., Crowl, G., Faheem, A.*, et al.* (2020). Sensitivity of nasopharyngeal swabs and saliva for the detection of severe acute respiratory syndrome coronavirus 2 (SARS-CoV-2). Clin Infect Dis.
10. Rao, M., Rashid, F.A., Sabri, F., Jamil, N.N., Zain, R., Hashim, R., Amran, F., Kok, H.T., Samad, M.A.A., and Ahmad, N. (2020). Comparing nasopharyngeal swab and early morning saliva for the identification of SARS-CoV-2. Clin Infect Dis.
11. Wyllie, A.L., Fournier, J., Casanovas-Massana, A., Campbell, M., Tokuyama, M., Vijayakumar, P., Warren, J.L., Geng, B., Muenker, M.C., Moore, A.J.*, et al.* (2020). Saliva or Nasopharyngeal Swab Specimens for Detection of SARS-CoV-2. N Engl J Med.
12. Robert Koch Institut. Nationale Teststrategie – wer wird in Deutschland auf das Vorliegen einer SARS-CoV-2 Infektion getestet? https://www.rki.de/DE/Content/InfAZ/N/Neuartiges_Coronavirus/Teststrategie/Nat-Teststrat.html. Download 2021-02-11.

13. Bundesministerium für Gesundheit. Verordnung zum Anspruch auf Testung in Bezug auf einen direkten Erregernachweis des Coronavirus SARS-CoV-2 (Coronavirus-Testverordnung – TestV). 27. Januar 2021.

14. Larremore DB, Wilder B, Lester E, et al. Test sensitivity is secondary to frequency and turnaround time for COVID-19 screening. *Sci Adv*. 2021;7(1):eabd5393. Published 2021 Jan 1. doi:10.1126/sciadv.abd5393.

15. Zhang K, Shoukat A, Crystal W, Langley JM, Galvani AP, Moghadas SM. Routine saliva testing for the identification of silent coronavirus disease 2019 (COVID-19) in healthcare workers [published online ahead of print, 2021 Jan 11]. *Infect Control Hosp Epidemiol*. 2021;1-5. doi:10.1017/ice.2020.1413.

16. Deckert et al. Effectiveness and cost-effectiveness of four different strategies for SARS-CoV-2 surveillance in the general population (CoV-Surv Study): a structured summary of a study protocol for a cluster-randomised, two-factorial controlled trial. Trials 2021. 22:39, https://doi.org/10.1186/s13063-020-04982-z.
