# Supplementary material for: Determining the optimal frequency of SARS-CoV-2 regular asymptomatic testing: A randomized feasibility trial in a home care setting
Source: PLoS One. 2024 Jul 3;19(7):e0303344. doi: 10.1371/journal.pone.0303344 (PMC11221670; doi:10.1371/journal.pone.0303344)
Supplement: S2 Protocol — (PDF) [file pone.0303344.s002.pdf]

Studienprotokoll

SuRIP/B-FAST

SARS-CoV-2

Risikobasierte Surveillance von Personen mit engem Kontakt zu Personen aus Risikogruppen im Rahmen der häuslichen Pflege im Raum Magdeburg in der SARS-CoV-2 Pandemie

Acronym: SuRIP

Eine prospektive, longitudinale, regionale Kohortenstudie zur Entwicklung von Surveillance-Konzepten und zur Überprüfung deren Akzeptanz bei pflegenden Personen/Angehörigen im häuslichen Bereich im Rahmen des bundesweiten Forschungsnetzes zur angewandten Surveillance und Testung (B-FAST).

SuRIP/ B-FAST

Studienprotokoll Version 1.0

15.02.2021

Studienleitung

Univ.Prof. Dr. med. Achim Kaasch

Institut für Medizinische Mikrobiologie und Krankenhaushygiene (IMMB)

Medizinische Fakultät der Otto-von-Guericke Universität Magdeburg

Leipziger Str. 44, 39120 Magdeburg

T +49 391 / 67 - 13392, F +49 391 / 67 - 13384

achim.kaasch@med.ovgu.de

Studienteam

Institut für Medizinische Mikrobiologie und Krankenhaushygiene (IMMB)

PD Dr. med. habil. Ina Tammer

Milica Dilas

Annett Hellriegel-Nehrkorn

Institut für Sozialmedizin und Gesundheitssystemforschung (ISMG)

Prof. Dr. Christian Apfelbacher, PhD

Robert Pohl, M.A.

Medizinische Fakultät der Otto-von-Guericke Universität Magdeburg

Modellierung: Max-Planck-Institut für Dynamik komplexer technischer Systeme Magdeburg

Prof. Dr. Peter Benner

Dr. Sara Grundel

Dr. Christian Himpe

Sandtorstr. 1

39106 Magdeburg

Zusammenfassung

**Titel**	Surveillance von pflegenden Angehörigen mit engem Kontakt zu Personen aus Risikogruppen im Raum Magdeburg in der SARS-CoV-2 Pandemie<br><br>Acronym: SuRIP - SARS-CoV-2<br><br>Eine prospektive, longitudinale, regionale Kohortenstudie zur Entwicklung von Surveillance-Konzepten und zur Überprüfung deren Akzeptanz bei pflegenden Personen/ Angehörigen im Rahmen des bundesweiten Forschungsnetzes zur angewandten Surveillance und Testung (B-FAST)
**Studienpopulation**	Angehörige, die Personen aus Risikogruppen (vulnerable Gruppen), wie Senioren oder Personen mit Behinderung pflegen
**Primäres Ziel**	Praktikabilität und Akzeptanz verschiedener Testfrequenzen eines einfachen, selbst durchführbaren und selbst auswertbaren Testsystems (Speicheltest) bei pflegenden Angehörigen.
**Sekundäre Ziele**	Gewinnung von Daten zur Modellierung der Testfrequenz.<br><br>Überprüfung der Testgüte (Sensitivität und Spezifität) von Selbsttest-Systemen.<br><br>Untersuchung des Einflusses externer Kontakte der pflegenden Personen (z. B. beruflich) und des aktuellen Infektionsgeschehens (aktuelle Inzidenz) und der damit verbundenen möglichen Risiken der Übertragung von SARS-CoV-2 auf die zu pflegende Person.<br><br>Messung der Impfakzeptanz und der Impfwilligkeit in dieser Personengruppe.

Studiendesign

Prospektive, longitudinale Pilot-Kohortenstudie mit serieller Selbsttestung mittels Speicheltest zunächst über einen Zeitraum von 6 Wochen. Es werden 45 pflegende Angehörige in 3 Gruppen mit verschiedenen Testfrequenzen randomisiert.

1. Gruppe: 1mal pro Woche
2. Gruppe: 2mal pro Woche
3. Gruppe: alle 2 Tage

Es werden jeweils 15 Personen jeder Gruppe per Zufall (Auslosung) zugeteilt.

Untersuchungsschema

Rekrutierung: Die Rekrutierung der Probanden erfolgt per Annonce in den lokalen Printmedien. Interessierte Personen melden sich telefonisch im Studiensekretariat.

Visite 0: Auswahl der Probanden entsprechend der festgelegten Ein– und Ausschlusskriterien (Mindestalter, pflegende Angehörige, externe Kontakten, schriftliche Einwilligungen, keine aktive COVID-19-Erkrankung; siehe Punkt 2.3. und 2.4.) bei erster telefonischer Kontaktaufnahme. Randomisierung der ausgewählten Probanden.

Visite 1: Persönlicher Besuch der Probanden im Studienzentrum, Entnahme eines kombinierten Nasen-Rachen-Abstriches zur PCR-Testung (Ausschluss eines SARS-CoV-2-Trägerstatus), Blutentnahme zur Antikörper-Bestimmung, Ausfüllen der Fragebögen „Erstkontakt“ und „Impfung“ durch die Studienteilnehmer/innen begleitet durch das Studienpersonal, Anleitung zur Selbsttestung und Dokumentation.

Visite 2-7: Wöchentliche telefonische Kontaktaufnahme (Einzelbefragungen) zur Abfrage der Testergebnisse, Abfrage des Fragebogens „Follow up“ zu Art und Häufigkeit von Kontakten in der letzten Woche sowie zu Veränderungen im beruflichen und privaten Umfeld, regelmäßige Einsendung der Salivetten für die PCR-Testung.

Visite 8: Abschluss-Visite, persönlicher Besuch der Studienteilnehmer/innen, Blutentnahme zur Antikörper-Bestimmung, Fragebogen „Abschlussgespräch“.

Teilnehmendes Zentrum

Universitätsmedizin Magdeburg

Finanzierung

Förderung durch das Bundesministerium für Bildung und Forschung im Rahmen des B-FAST- Projektes des Nationales Forschungswerk der Universitätsmedizin zu Covid-19.

1 Einleitung

Ende 2019 trat ein neues Coronavirus SARS-CoV-2 (severe acute respiratory syndrome coronavirus type 2) erstmals in China als Verursacher von Lungenentzündungen auf. Im Januar 2020 wurde das Virus durch die chinesischen Behörden identifiziert und an die WHO gemeldet. Seitdem breitet sich das neue Coronavirus weltweit aus. Im März 2020 wurde der Ausbruch offiziell durch den WHO-Generalsekretär zu einer Pandemie erklärt (1).

Coronaviren verursachen beim Menschen vorwiegend milde Erkältungskrankheiten, können aber auch schwere Lungenentzündungen hervorrufen. SARS-CoV-2 verursacht die Erkrankung COVID-19. COVID-19 kann sich in vielfältiger Weise und nicht nur in der Lunge, sondern auch in anderen Organsystemen, wie dem Nervensystem, dem Gastrointestinaltrakt, dem Herz-Kreislaufsystem oder in den Nieren manifestieren. Darüber hinaus ist eine relativ große Bandbreite an dermatologischen Manifestationen beschrieben, die jedoch insgesamt selten sind (0,2-1,2%). Einige Patienten mit schwerer SARS-CoV-2-Infektion entwickeln 8-15 Tage nach Erkrankungsbeginn eine Verschlechterung im Sinne eines Hyperinflammationssyndroms, in dessen Folge es zu Multiorganversagen kommen kann, dass mit einer hohen Mortalität assoziiert ist. Schwere Verläufe können auch bei Personen ohne bekannte Vorerkrankung und bei jüngeren Patienten auftreten. Bei bestimmten Personengruppen werden schwere Krankheitsverläufe jedoch deutlich häufiger beobachtet. Zu diesen sogenannten Risikogruppen oder vulnerable Gruppen gehören vor allem ältere Personen. 86% der in Deutschland an COVID-19 Verstorbenen waren 70 Jahre alt oder älter (Altersmedian: 82 Jahre) (2).

Mit zunehmenden Alter steigt der Anteil pflegebedürftiger Personen. Nach Angaben des Statistischen Bundesamtes waren Ende 2019 4,13 Mio. Menschen in Deutschland pflegebedürftig im Sinne des Pflegeversicherungsgesetzes (SGB XI). Vier von fünf Pflegebedürftigen (80 % beziehungsweise 3,31 Millionen) wurden zu Hause versorgt. Davon wurden 2,33 Millionen Pflegebedürftige überwiegend durch Angehörige gepflegt (3). Die pflegenden Angehörigen haben in aller Regel nicht nur einen engen Kontakt zu der zu pflegenden Person, sondern häufig auch andere externe Kontakte zum Beispiel durch deren berufliche Tätigkeit, wodurch ein potentielles Risiko zur Infektion und Übertragung auf die zu Pflegenden besteht.

Zudem breiten sich auch in Deutschland neue SARS-CoV-2- Varianten aus, bei denen man die Auswirkungen noch nicht einschätzen kann. Seit Mitte Dezember 2020 wird aus dem Vereinigten Königreich (VK) über die zunehmende Verbreitung der SARS-CoV-2 Linie B.1.1.7 (VOC 202012/01; VOC: variant of concern) berichtet, die sich durch eine ungewöhnlich hohe Zahl an Mutationen insbesondere im viralen Spike (S)-Protein auszeichnet, mit deren Hilfe das Virus an die menschlichen Zellen andockt. Man geht mittlerweile davon aus, dass diese Variante eine erhöhte Übertragbarkeit aufweist, die in einer höheren Reproduktionszahl resultiert (2).

Eine Herdenimmunität zum Schutz vor COVID-19 kann entweder durch eine hohe Impfquote oder durch eine natürliche Infektion erreicht werden. Eine Herdenimmunität durch natürliche Infektion würde voraussetzen, dass eine große Bevölkerungsgruppe (mehr als die Hälfte) die Erkrankung durchmacht und Antikörper bildet. Es ist allerdings noch nicht geklärt, ob nach durchgemachter Infektion jeder eine volle Immunität erlangt und wie lange diese anhält. Dieses Szenario würde mit einer hohen Zahl an Schwerstkranken und Todesfällen einhergehen und wäre ethisch nicht vertretbar (4).

Der einzige derzeit gangbare Weg zur Erreichung einer ausreichenden Herdenimmunität ist die Impfung. Zurzeit sind in Deutschland 3 Impfstoffe zugelassen. Aufgrund des hohen Bedarfs können diese jedoch noch nicht ausreichenden Mengen zur Verfügung gestellt werden. Laut Angaben des RKI sind in Sachsen-Anhalt derzeit nur 1,5% der erwachsenen Bevölkerung voll immunisiert (2 Impfungen) (5). Erschwerend kommt hinzu, dass laut Empfehlung der STIKO (Ständige Impfkommission am Robert Koch-Institut) der Vektor-basierte COVID-19-Impfstoff von AstraZeneca aktuell aufgrund der derzeit verfügbaren Daten nur für Personen im Alter von

18 bis 64 Jahren empfohlen wird. Aus diesen Gründen hat das RKI prioritär zu impfende Risikogruppen definiert, die eine besonders hohe Vulnerabilität oder ein besonders hohes Expositionsrisiko haben (Stufe 1=höchste Priorität). Da die meisten pflegenden Angehörigen wahrscheinlich am ehesten der Stufe 3 oder einer höheren Stufe in der Priorisierungsliste der STIKO zuzuordnen sind, werden sie erst mit zeitlicher Verzögerung von dieser Schutzmaßnahme profitieren (6).

Wichtige Maßnahmen zur Eindämmung der Pandemie sind die Einhaltung der AHA-L-Regeln (Abstand-Händewaschen-Alltagsmaske-Lüften), Quarantänemaßnahmen bei Infektion oder Kontakt zu einem Infizierten und die Testung bei Verdacht auf Infektion (7=Testkriterien). Als Goldstandard für die Testung gilt nach wie vor die PCR aus Nasopharynx-Abstrichen, Rachenabstrichen oder kombinierten Nasen-Rachen-Abstrichen. Die Verwendung anderer Probenmaterialien, wie z.B. von Rachenspülwasser/Gurgelwasser und Speichel wird diskutiert, da die Gewinnung dieser Materialien deutlich besser toleriert wird. Allerdings liegen für diese Materialien deutlich weniger Erfahrungswerte vor. Für Speichel beschreiben einige Gruppen eine geringere klinisch-diagnostische Sensitivität (8, 9), während andere Gruppen vergleichbare bzw. im Fall einiger Studien auch eine höhere Sensitivität der PCR-Diagnostik im Vergleich zum beiderseitigen Nasopharynxabstrich feststellten (10, 11).

Antigenteste werden zunehmend zur Entlastung der PCR-Diagnostik eingesetzt. In der „Nationalen Teststrategie SARS-CoV-2“ des RKI wird empfohlen, Personal z.B. in Pflegeeinrichtungen und in der ambulanten Pflege ohne COVID-19-Fall in Gebieten mit erhöhter 7-Tage-Inzidenz (>50/100.000 Einwohner) regelmäßig z.B. mittels Antigentest zu testen. Für eine regelmäßige Reihen-Testung sieht die Testverordnung einen Anspruch auf Testung einmal in der Woche vor (12,13).

Aufgrund fehlender Studien und einer möglicherweise niedrigeren Sensitivität wird von Reihenuntersuchungen asymptomatischer Personen derzeit abgeraten, da die Testergebnisse nur eine Momentaufnahme darstellen. Modellierungen und erste bevölkerungsbasierte Studien legen jedoch nahe, dass für eine effektive Surveillance vor allem die Testfrequenz und die Schnelligkeit, mit der ein Ergebnis zur Verfügung steht, von Bedeutung sind und weniger die Sensitivität des Testsystems (14, 15 ,16).

1.1 Notwendigkeit einer Studie

Obwohl pflegende Angehörige einen sehr hohen Anteil an der Pflege vulnerabler Personen haben und daher auch der Schutz dieser Gruppe sehr wichtig ist, gibt es bisher keine Empfehlungen für eine Testung dieser Personengruppe. Ebenfalls unklar ist, wie hoch derzeit der Anteil der Personen mit einer durchgemachten Infektion ist, wie viele Personen bereits geimpft sind und wie die generelle Einstellung zur Impfung in dieser Bevölkerungsgruppe ist.

Die Erhebung dieser Daten ist erforderlich, um effektive Surveillance-Konzepte für diese Bevölkerungsgruppe entwickeln zu können, welche wiederum den Schutz und die Sicherheit des zu betreuenden Personenkreises erhöhen.

1.2 Ziele

Primäres Ziel der Studie ist es, die Praktikabilität und Akzeptanz verschiedener Testfrequenzen eines einfachen, selbst durchführbaren und selbst auswertbaren Testsystems (Speicheltest) in der bisher unterrepräsentierten Bevölkerungsgruppe der pflegenden Angehörigen zu ermitteln.

Wichtige **sekundäre Ziele** sind:

1. Gewinnung von Daten zur Modellierung der Testfrequenz, soziodemografischer Daten zur Verbesserung mathematischer Modelle und zur Entwicklung realitätsnaher Surveillancesysteme.

2. Überprüfung der Testgüte (Sensitivität und Spezifität) von Selbsttest-Systemen.
3. Untersuchung des Einflusses externer Kontakte der pflegenden Personen (z. B. beruflich) und des aktuellen Infektionsgeschehens (aktuelle Inzidenz) und der damit verbundenen möglichen Risiken der Übertragung von SARS-CoV-2 auf die zu pflegende Person.
4. Messung der Impfakzeptanz und der Impfwilligkeit in dieser Personengruppe.

2 Methoden

2.1 Design Pilotstudie

Prospektive, longitudinale Kohortenstudie mit serieller Selbsttestung mit einem Speichel-Test (selbstdurchführbarer Antigen-Schnelltest aus Speichelproben) als Pilotphase über einen Zeitraum von 6 Wochen an 45 Personen. Folgende Frequenzen der Selbsttestung werden untersucht:

Gruppe: 1mal pro Woche

Gruppe: 2mal pro Woche

Gruppe: alle 2 Tage

Es werden jeweils 15 Personen jeder Gruppe per Zufall zugeteilt. Die Zuteilung erfolgt über Lose in vorgefertigten Umschlägen, die bei der ersten Visite geöffnet werden.

Die Pilotphase mit 45 Personen soll im März 2021 starten (vorgesehener Untersuchungszeitraum 15.03.2021 bis 30.04.2021).

Bei Erstkontakt (Visite 1) wird den Probanden/innen das Studiendesign in einem persönlichen Gespräch ausführlich erklärt. Es erfolgt ebenfalls eine Aufklärung zur Blutentnahme und zur Entnahme eines Nasen-Rachen-Abstriches zur PCR-Testung.

Nach schriftlicher Einwilligung der Probanden/Probandinnen erfolgt eine Blutentnahme zum Nachweis bereits vorhandener Antikörper gegen SARS-CoV-2 als Hinweis auf eine durchgemachte Infektion sowie die Entnahme eines kombinierten Rachen-Nasen-Abstrichs zur Abklärung eines SARS-CoV-2-Trägerstatus (Infektion) durch den Studienarzt. Die Ergebnisse werden den Probanden/innen zeitnah telefonisch und schriftlich mitgeteilt.

Bei dieser Visite werden die Probanden/innen zur Durchführung des Speicheltestes angeleitet, den sie einmal selbstständig vor Ort durchführen. Anschließend wird den Probanden/innen die Auswertung des Testes erklärt. Zusätzlich erfolgt eine Anleitung zur Durchführung der Speichelentnahme mit dem Salivetten®-System. Sie führen diesen Test ebenfalls einmal selbstständig vor Ort durch.

Ergänzend zu den jeweiligen Tests werden den Probanden/innen zwei Fragebögen (Fragebogen „Erstkontakt“ und „Impfungen“) vorgelegt, die sie im Studienzentrum zusammen mit dem Studienpersonal ausfüllen sollen.

Am Ende des ersten Gesprächs werden jedem/jeder Probanden/innen folgende Materialien und Unterlagen ausgehändigt:

- entsprechende Anzahl von Speicheltests (je nach Randomisierungsgruppe)
- entsprechende Anzahl von Salivetten
- schriftliche Anleitung zum Speicheltest
- Formular für die Dokumentation der Testergebnisse (Formblätter zur Dokumentation der Selbst- Test-Ergebnisse, Appendix 7.4.)
- frankierte Briefumschläge zur Rücksendung der Salivetten

- Einwilligungserklärung zur Teilnahme in Kopie (Appendix 7.2)
- Informationsblatt zur Teilnahme an der Studie (Teilnehmeraufklärung, Appendix 7.1)

In den folgenden Wochen sollen der Probanden/innenden Schnelltest allein zu Hause durchführen und das Testergebnis in einen Dokumentationsbogen eintragen. Zusätzlich werden die Probanden/innengebeten, wenn möglich die Testkartuschen mit dem Handy zu fotografieren und die Fotos zum Abschlussbefragung mitzubringen. Außerdem sollen die Studienteilnehmer/innen nach der Durchführung des Speicheltestes Speichel mit dem Salivetten-System sammeln und das Probenröhrchen per Post an das Studienzentrum schicken.

Die Probanden/Probandinnen werden 1 x wöchentlich telefonisch vom Studienzentrum kontaktiert (Einzelbefragungen), um mithilfe eines strukturierten Fragebogens (Fragebogen „Follow up") mögliche Veränderungen aus der Erstbefragung (Fragen zu persönlichen Kontakten, Fragen zu Veränderungen im beruflichen und privaten Umfeld, etc.) und aktuelle Erhebungen zur Akzeptanz und Praktikabilität der Schnelltests und Salivetten-Systeme wöchentlich zu erfassen. Gleichzeitig wird das Ergebnis der Schnellteste erfragt und ein Termin für die nächste telefonische Befragung vereinbart.

Am Ende des Untersuchungsintervalls erfolgt ein persönliches Abschlussgespräch im Studienzentrum, bei dem mithilfe eines strukturierten Fragebogens (Fragebogen „Abschlussgespräch") nochmals Daten zur Akzeptanz und zur Praktikabilität sowie Verbesserungsvorschläge seitens der Probanden/Probandinnen zur Studie erfasst werden. Es wird eine zweite Blutentnahme für eine erneute Antikörpertestung durchgeführt, um kürzer zurückliegende Infektionen zu erfassen.

Rekrutierung der Teilnehmer

Die Rekrutierung der Probanden/Probandinnen erfolgt per Annonce in den lokalen Printmedien. Interessierte Personen melden sich telefonisch Studiensekretariat.

Nachrekrutierung

Verlassen Probanden/Probandinnen in der ersten Woche die Studie, erfolgt eine Nachrekrutierung. Dazu werden Interessenten, die sich auf die Annonce gemeldet haben, aber nicht bei der Erstrekrutierung berücksichtigt werden konnten, telefonisch kontaktiert.

2.2 Zeitplan

<u>Meilensteine:</u>

19.02.2021<br>Ethikeinreichung	15.03.2021<br>Start Pilotphase<br>(6 Wochen)	15.06.2021<br>Ende<br>Datensammlung	01.08.2021<br>Ende der<br>Datenauswertung

Pilotstudie

	Jan	Feb	März	April	Mai	Juni	Juli	Aug	Sep
Vorbereitung									
Rekrutierung									
Studien-einschluss									
Studien-periode									
Studien-abschluss									

2.3 Einschlusskriterien

- Mindestalter von 18 Jahren
- Pflegende Angehörige mit mindestens 2 Kontakten pro Woche von mindestens 30 Minuten Dauer zu der zu pflegenden Person
- Pflegende Angehörige haben externe Kontakte (z.B. Berufstätigkeit)
- schriftliche Einwilligung zur Studienteilnahme.
- Schriftliche Einwilligung zur Blutentnahme (2x) und zur Entnahme eines Nasen-Rachen-Abstriches (1x)

2.4 Ausschlusskriterien

- aktive COVID-19-Erkrankung

2.5 Verfahren bei Rückzug der Einwilligung

Wenn ein/e Proband/Probandin seine Einwilligung zur Studienteilnahme widerruft, so muss dies schriftlich erfolgen. Der/die Proband/Probandin erhält dann eine schriftliche Bestätigung des Eingangs. Die bis dahin erhobenen Daten werden ausgewertet.

2.6 Verfahren bei Auftreten einer COVID-19-Infektion während der Studie

Die Probanden/Probandinnen werden darüber aufgeklärt, dass bei Auftreten von Erkältungssymptomen umgehend Maßnahmen zur Distanzierung ergriffen und der Hausarzt

kontaktiert werden muss. Über die Abklärung einer akuten COVID-19-Erkrankung entscheidet der Hausarzt. Alternativ wird den Probanden/Probandinnen eine Testung mittels PCR in der Fieberambulanz des Universitätsklinikums vermittelt. Die Probanden und Probandinnen werden darüber aufgeklärt, dass der Selbsttest diese Maßnahmen nicht ersetzt.

Wird eine COVID-19-Infektion nachgewiesen, müssen die Probanden/Probandinnen das Studienzentrum informieren. Sie werden dann aus der Studie ausgeschlossen.

2.7 Verfahren bei positivem Speicheltest

Wird der Speicheltest positiv, muss der/die Proband/ Probandin umgehend telefonisch Kontakt mit dem Studienzentrum aufnehmen.

Zum Ausschluss einer COVID-19-Infektion wird ein kombinierter Rachen-Nasen-Abstrich durch geschultes Personal im Studienzentrum oder in der Fieberambulanz am Universitätsklinikum Magdeburg durchgeführt werden und eine PCR-Untersuchung auf SARS-CoV-2 erfolgen.

Bei negativem PCR-Ergebnis ist eine Infektion zum Zeitpunkt der Probenentnahme ausgeschlossen. Der Proband/ Probandin erhält einen Selbstbeobachtungsbogen, der täglich auszufüllen ist, und verbleibt zunächst in der Studie. Stellen sich Symptome einer COVID-19-Infektion ein, muss das Studienzentrum informiert und der Hausarzt kontaktiert werden (siehe Punkt 2.6).

Bei positiven PCR-Ergebnis ist eine SARS-CoV-2-Infektion bestätigt. Der/ die Proband/ Probandin muss sich dann in häusliche Isolation begeben. Das Ergebnis der PCR-Testung wird dem Gesundheitsamt mitgeteilt, welches werden weitere Maßnahmen veranlasst.

2.8 Verfahren bei Tod der zu pflegenden Person

Verstirbt die zu pflegende Person im Verlauf der Studie, gehört der Teilnehmer nicht mehr zu der untersuchten Personengruppe und wird aus der Studie ausgeschlossen. Die ausgehändigten und bis zu diesem Zeitpunkt noch nicht genutzten Speicheltests darf der Proband behalten.

2.9 Datenerhebung und Datenfluss

Datenerhebung:

Es werden 2 Visiten mit persönlicher Vorstellung im Studienzentrum durchgeführt. Die anderen Kontakte erfolgen telefonisch in Form von Einzelbefragungen.

<u>Visitenplan</u>

	**Visite 1 Einschluss**	**Visite 2 Woche 1**	**Visite 3 Woche 2**	**Visite 4 Woche 3**	**Visite 5 Woche 4**	**Visite 6 Woche 5**	**Visite 7 Woche 6**	**Visite 8 Abschluss**
Besuch	✕							✕
Antikörper-Bestimmung Blutentnahme 10ml Serum	✕							✕
PCR-Testung/ Nase-Rachen-Abstrich	✕							
Fragebogen	✕							✕
Randomisierung	✕							

Selbsttest durch Teilnehmer (Speicheltest, Salivetten)	×	×	×	×	×	×	×	
Abfrage Testergebnis (telefonisch) Befragung		×	×	×	×	×	×	
Fragebogen (telefonisch) Befragung		×	×	×	×	×	×	

Die Erfassung der Erstbefragung, der Impfakzeptanz und der Impfwilligkeit erfolgt pseudonymisiert mit Papierfragebögen während des Erstgesprächs im Studienzentrum und wird während der Pilotphase seitens des IMMB aufbewahrt und zur späteren Auswertung an das Institut für Sozialmedizin und Gesundheitssystemforschung (ISMG) der Universitätsmedizin Magdeburg übermittelt. Die telefonische Befragung der Follow-up Fragebögen wird von Mitarbeiterinnen des IMMB durchgeführt und ebenfalls nach Abschluss der Pilotstudie an das ISMG in pseudonymisierter Form weitergegeben.

Datenspeicherung

Pseudonymisierte Blutanalyseergebnisse: Labor-EDV-System der Universitätsmedizin Magdeburg.

Pseudonymisierte Befragungsergebnisse: Die Befragungsdaten werden aus Fragebögen (sowohl telefonisch als auch in Papierform) im ISMG gespeichert. Die Datenspeicherung erfolgt pseudonymisiert.

Randomisierung und Pseudonymisierung

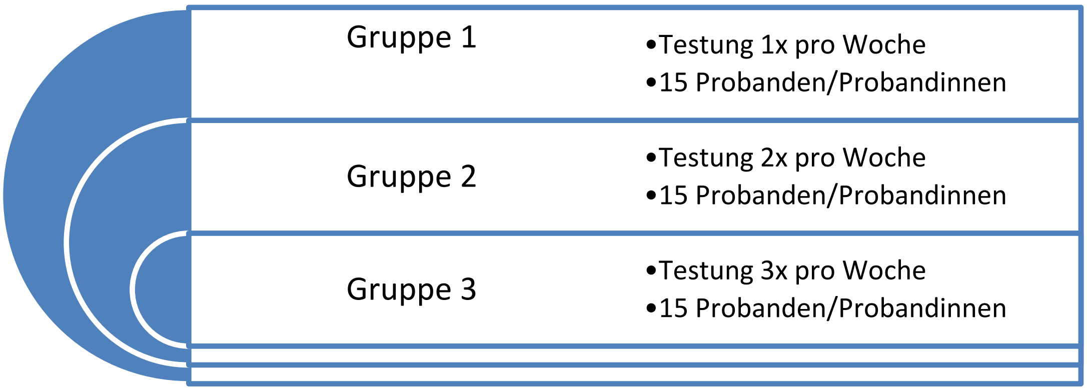

Abb.1

In einem Los-Verfahren ziehen die Probanden/Probandinnen einen neutralen Umschlag, welcher die Teilnehmer-Nummer und damit die jeweilige Gruppen-Zuordnung enthält (Abb. 1).

Datenfluss

Eine systematische Übersicht zur Datenerhebung und zum Datenfluss ist im Appendix unter 7.5. aufgeführt

Die von den Probanden/Probandinnen ausgefüllten Fragebögen werden mit einem Teilnehmerpseudonym (TN-ID) seitens des IMMB versehen. Selbiges geschieht bei der telefonischen Befragung der Follow-up Fragebögen seitens der Befragenden Person. Diese TN-ID bleibt über den Zeitraum der Pilotstudie für jede/n Teilnehmer/in gleich.

Aus den Antikörperbefunden wird der Antikörperstatus (Testergebnisse) extrahiert und zusammen mit der TN-ID separat gespeichert.

Die Ergebnisse des PCR-test,- Antikörper-Tests, und der Speicheltests werden mit der zugehörigen TN-ID an das ISMG übermittelt und dort bis zur Beendigung der Studie gespeichert.

Im ISMG werden die Befragungsdaten zusammen mit den Daten zu den Tests über die TN-ID verknüpft. Der Datensatz wird als Auswertedatensatz gespeichert.

Es folgt die Analyse des Auswertedatensatzes durch die Wissenschaftler/innen der Studie.

2.10 Laboruntersuchungen

2.10.1 Probenentnahme

Bei dem Erstkontakt erfolgt eine Blutentnahme von 10ml (Serummonovette) durch periphere Venenpunktion und ein Mund-Nasen-Abstrich für die RT-qPCR in der Ambulanz des IMMB (Personal-Screening-Ambulanz).

Die Probanden/Probandinnen führen unter Anleitung den ersten Speicheltest im Studienzentrum durch. Dazu soll der COVID-19 Antigen Saliva-Test Card, Fa. ultimed Products Deutschland GmbH, Ahrensburg, Deutschland genutzt werden. Die ausreichende Verfügbarkeit der Testkits wird vor Beginn der Studie geprüft.

Die weiteren Speichelteste werden selbstständig zu Hause durchgeführt. Die Ablesung des Testergebnisses erfolgt selbstständig durch die Studienteilnehmer/innen. Die Testergebnisse werden in einem Dokument festgehalten und wenn möglich fotografiert.

Parallel zu dem Speicheltest sammeln die Studienteilnehmer/innen Speichel mit dem Salivetten-System (Salivette®, Fa. Sarstedt, Nümbrecht, Deutschland). Das Probenröhrchen wird in einem frankierten Briefumschlag an das Studienzentrum verschickt.

Die Proben werden über das Labor-EDV-System des IMMB mit der Studiennummer (Pseudonym) erfasst und 30 Jahre lang gespeichert.

Serologische und molekularbiologische Untersuchungen

Zur Bestimmung von Antikörpern gegen SARS-CoV-2 wird der LIAISON SARS-CoV-2 TrimericS IgG Assay (Fa. DiaSorin) verwendet. Bei positivem Antikörper-Befund wird eine Testung mit den „ImmuSAFE™ Covid+“ System (Fa. Aicone) durchgeführt zur Abgrenzung von Antikörpern gegen endemische Coronaviren.

Die Bestimmung von SARS-CoV-2-RNA erfolgt mittels kommerzieller Testsysteme im diagnostischen Bereich des Instituts für Medizinische Mikrobiologie und Krankenhaushygiene.

2.10.2 Probenaufbewahrung

Alle Proben werden bei -80°C im IMMB für 2 Jahre aufbewahrt.

2.11 Ethische Überlegungen

2.11.1 Einwilligung

Es erfolgt eine schriftliche Einwilligungserklärung (Appendix 7.2.) Darin wird auf die Zielsetzung und die Inhalte der Studie, die Messinstrumente, die Art und der Inhalt der Datenerhebung und -speicherung hingewiesen sowie auf die Freiwilligkeit der Teilnahme und der Möglichkeit des jederzeit möglichen Widerrufs der Einwilligung. Den Teilnehmer/innen entstehen keine Nachteile bei einer Nichtteilnahme. Den Teilnehmer/innen wird vor ihrer Einwilligung/Verweigerung ausreichend Zeit gegeben, ihre Entscheidung zu bedenken. Für Rückfragen stehen die Mitarbeiter/innen des Institutes für Medizinische Mikrobiologie und Krankenhaushygiene zur Verfügung.

2.11.2 Risiken und Vorteile

Es wird bei Erstkontakt Blut durch ärztliches bzw. medizinisches Personal abgenommen. Bei der diagnostischen Blutabnahme besteht keine nennenswerte Infektions- oder Verletzungsgefahr. Eventuell kann es zu psychisch ausgelösten Kreislaufproblemen kommen, in sehr seltenen Fällen zum Schock. Relativ häufig entwickelt sich aber an der Punktionsstelle ein blauer Fleck. In seltenen Fällen kann es bei einem Rachen-Nasen-Abstrich zu einer Verletzung der Schleimhaut oder zum Auslösen eines Brechreizes kommen. Für die Probanden/ Probandinnen ergeben sich weder direkte Vorteile noch Nachteile. Sie erhalten Zugang zu Selbsttests (kostenlos zur Verfügung gestellte Test-Kits) und Antikörpertestung.

2.11.3 Datenschutz

Die Datenverarbeitung (Erhebung, Speicherung, Nutzung) im Rahmen der SuRIP-Studie erfolgt auf Basis der Datenschutzkonzepte der Universitätsmedizin Magdeburg. Die Erfassung der Datenerhebung im Rahmen der Befragung (telefonisch) erfolgt passwortgeschützt auf dem Server des IMMB) der. Die Datenschutzkonzepte gehen konform mit der EU-Datenschutzgrundverordnung (EU-DSGVO) und dem Bundesdatenschutzgesetz.

Datenspeicherorte:

Personenidentifizierende Daten: Patienteninformationssystem des IMMB, passwortgeschützt

Pseudonymisierte Befragungsergebnisse: permanent im IMMB

Datenzugang:

Der Datenzugang zu den Studiendaten ist nur für die an der Studie beteiligten Mitarbeiter/innen im IMMB und möglich. Eine Namensliste mit der Teilnehmer-Indifikationsnummer (TN-ID) wird beim IMMB passwortgeschützt gespeichert.

Die Verknüpfung mit personenidentifizierenden Daten aus dem Patienteninformationssystem über die ID-TN ist ausgeschlossen.

Pseudonymisierung:

In einem Los-Verfahren ziehen die Probanden/Probandinnen einen neutralen Umschlag, welcher die Teilnehmer-Nummer und damit die jeweilige Gruppen-Zuordnung enthält.

2.11.4 Sicherheit des Personals

Der Schutz vor COVID-19 Infektionen erfolgt nach den jeweils gültigen Hygienerichtlinien der UMMD.

2.12 Finanzierung

Im Rahmen des „Bundesweiten Forschungsnetz Angewandte Surveillance und Testung“ gefördert.

3 Statistische Analyse

Für die statistische Analyse der Daten sind deskriptive Auswertungen geplant.

3.1 Epidemiologische Indikatoren

Es werden innerhalb der Gruppe von pflegenden Angehörigen Häufigkeiten einer SARS-COV 19 Infektion sowie die Surveillance untersucht und differenziert entsprechend der soziodemografischen Merkmale und Kontakthäufigkeiten.

4 Kommunikation der Ergebnisse

Die Ergebnisse sollen über wissenschaftliche Publikationen in internationalen und nationalen Journals mit Peer-Review-Verfahren im Open Access frei zugänglich gemacht werden. Ggf. werden Ergebnisse durch Vorträge & Poster bei nationalen und internationalen Fachkongressen, sowie durch Präsentationen auf Veranstaltungen kommuniziert.

5 Literatur

1. https://www.euro.who.int/de/health-topics/health-emergencies/coronavirus-covid-19/novel-coronavirus-2019-ncov. WHO2021 abgerufen 09.02.2021

2. Robert Koch Institut. Epidemiologischer Steckbrief zu SARS-CoV-2 und COVID-19. https://www.rki.de/DE/Content/InfAZ/N/Neuartiges_Coronavirus/Steckbrief.html;jsessionid=A4CD5057E0CB43880DA27789570EC7D1.internet052?nn=2386228, download 2021-02-11)

3. Statistisches Bundesamt (Destatis). Pflegestatistik 2019. Deutschlandergebnisse. https://www.destatis.de/DE/Themen/Gesellschaft-Umwelt/Gesundheit/Pflege/_inhalt.html, download 2021 02 11.

4. Papachristodoulou E, Kakoullis L, Parperis K, Panos G. Long-term and herd immunity against SARS-CoV-2: implications from current and past knowledge. Pathog Dis. 2020;78(3):ftaa025. doi:10.1093/femspd/ftaa025.

5. Robert Koch Institut. Digitales Impfquotenmonitoring zur COVID-19-Impfung. https://www.rki.de/DE/Content/InfAZ/N/Neuartiges_Coronavirus/Daten/Impfquoten-Tab.%20html. download 2021-02-11.
6. STIKO-Empfehlung zur COVID-19-Impfung, Epidemiologisches Bulletin 5 | 2021 4. Februar 2021
7. Robert Koch Institut. Testkriterien für die SARS-CoV-2 Diagnostik bei symptomatischen Patienten mit Verdacht auf COVID-19. https://www.rki.de/DE/Content/InfAZ/N/Neuartiges_Coronavirus/Teststrategie/Testkriterien_Herbst_Winter.html. Download 2021-02-21.

8. Chen, J.H., Yip, C.C., Poon, R.W., Chan, K.H., Cheng, V.C., Hung, I.F., Chan, J.F., Yuen, K.Y., and To, K.K. (2020). Evaluating the use of posterior oropharyngeal saliva in a point-of-care assay for the detection of SARS-CoV-2. Emerg Microbes Infect *9*, 1356-1359.
9. Jamal, A.J., Mozafarihashjin, M., Coomes, E., Powis, J., Li, A.X., Paterson, A., Anceva-Sami, S., Barati, S., Crowl, G., Faheem, A.*, et al.* (2020). Sensitivity of nasopharyngeal swabs and saliva for the detection of severe acute respiratory syndrome coronavirus 2 (SARS-CoV-2). Clin Infect Dis.
10. Rao, M., Rashid, F.A., Sabri, F., Jamil, N.N., Zain, R., Hashim, R., Amran, F., Kok, H.T., Samad, M.A.A., and Ahmad, N. (2020). Comparing nasopharyngeal swab and early morning saliva for the identification of SARS-CoV-2. Clin Infect Dis.
11. Wyllie, A.L., Fournier, J., Casanovas-Massana, A., Campbell, M., Tokuyama, M., Vijayakumar, P., Warren, J.L., Geng, B., Muenker, M.C., Moore, A.J.*, et al.* (2020). Saliva or Nasopharyngeal Swab Specimens for Detection of SARS-CoV-2. N Engl J Med.
12. Robert Koch Institut. Nationale Teststrategie – wer wird in Deutschland auf das Vorliegen einer SARS-CoV-2 Infektion getestet? https://www.rki.de/DE/Content/InfAZ/N/Neuartiges_Coronavirus/Teststrategie/Nat-Teststrat.html. Download 2021-02-11.

13. Bundesministerium für Gesundheit. Verordnung zum Anspruch auf Testung in Bezug auf einen direkten Erregernachweis des Coronavirus SARS-CoV-2 (Coronavirus-Testverordnung – TestV). 27. Januar 2021.

14. Larremore DB, Wilder B, Lester E, et al. Test sensitivity is secondary to frequency and turnaround time for COVID-19 screening. *Sci Adv*. 2021;7(1):eabd5393. Published 2021 Jan 1. doi:10.1126/sciadv.abd5393.

15. Zhang K, Shoukat A, Crystal W, Langley JM, Galvani AP, Moghadas SM. Routine saliva testing for the identification of silent coronavirus disease 2019 (COVID-19) in healthcare workers [published online ahead of print, 2021 Jan 11]. *Infect Control Hosp Epidemiol*. 2021;1-5. doi:10.1017/ice.2020.1413.

16. Deckert et al. Effectiveness and cost-effectiveness of four different strategies for SARS-CoV-2 surveillance in the general population (CoV-Surv Study): a structured summary of a study protocol for a cluster-randomised, two-factorial controlled trial. Trials 2021. 22:39, https://doi.org/10.1186/s13063-020-04982-z.
